# Influence of Four Veterinary Antibiotics on Constructed Treatment Wetland Nitrogen Transformation

**DOI:** 10.3390/toxics12050346

**Published:** 2024-05-08

**Authors:** Matthew V. Russell, Tiffany L. Messer, Deborah A. Repert, Richard L. Smith, Shannon Bartelt-Hunt, Daniel D. Snow, Ariel P. Reed

**Affiliations:** 1 Biosystems and Agricultural Engineering Department, University of Kentucky, 128 Barnhardt, Lexington, KY 40506, USA; tiffany.messer@uky.edu; 2 United States Geological Survey, Water Resources Mission Area, 3215 Marine St., Boulder, CO 80303, USA; darepert@usgs.gov (D.A.R.); rlsmith@usgs.gov (R.L.S.); apzreed@gmail.com (A.P.R.); 3 Department of Civil and Environmental Engineering, University of Nebraska-Lincoln, Lincoln, NE 68508, USA; sbartelt@unl.edu; 4 School of Natural Resources, East Campus, University of Nebraska-Lincoln, 101 Hardin Hall, Lincoln, NE 68583, USA; dsnow1@unl.edu; 5 Water Sciences Laboratory, East Campus, University of Nebraska-Lincoln, 1840 N. 37th Street, Lincoln, NE 68583, USA

**Keywords:** wetlands, nitrogen, nitrification, denitrification, veterinary antibiotics

## Abstract

The use of wetlands as a treatment approach for nitrogen in runoff is a common practice in agroecosystems. However, nitrate is not the sole constituent present in agricultural runoff and other biologically active contaminants have the potential to affect nitrate removal efficiency. In this study, the impacts of the combined effects of four common veterinary antibiotics (chlortetracycline, sulfamethazine, lincomycin, monensin) on nitrate-N treatment efficiency in saturated sediments and wetlands were evaluated in a coupled microcosm/mesocosm scale experiment. Veterinary antibiotics were hypothesized to significantly impact nitrogen speciation (e.g., nitrate and ammonium) and nitrogen uptake and transformation processes (e.g., plant uptake and denitrification) within the wetland ecosystems. To test this hypothesis, the coupled study had three objectives: 1. assess veterinary antibiotic impact on nitrogen cycle processes in wetland sediments using microcosm incubations, 2. measure nitrate-N reduction in water of floating treatment wetland systems over time following the introduction of veterinary antibiotic residues, and 3. identify the fate of veterinary antibiotics in floating treatment wetlands using mesocosms. Microcosms containing added mixtures of the veterinary antibiotics had little to no effect at lower concentrations but stimulated denitrification potential rates at higher concentrations. Based on observed changes in the nitrogen loss in the microcosm experiments, floating treatment wetland mesocosms were enriched with 1000 μg L^−1^ of the antibiotic mixture. Rates of nitrate-N loss observed in mesocosms with the veterinary antibiotic enrichment were consistent with the microcosm experiments in that denitrification was not inhibited, even at the high dosage. In the mesocosm experiments, average nitrate-N removal rates were not found to be impacted by the veterinary antibiotics. Further, veterinary antibiotics were primarily found in the roots of the floating treatment wetland biomass, accumulating approximately 190 mg m^−2^ of the antibiotic mixture. These findings provide new insight into the impact that veterinary antibiotic mixtures may have on nutrient management strategies for large-scale agricultural operations and the potential for veterinary antibiotic removal in these wetlands.

## 1. Introduction

Nitrate-N (NO_3_-N) is a common water contaminant worldwide and a major cause of drinking water impairment across the United States [1,2]. Although necessary for plant growth, acute and chronic NO_3_-N exposures exceeding recommended drinking water standards (10 mg L^−1^ NO_3_-N) can be detrimental to human and environmental health [3,4,5]. In humans, NO_3_-N exposure can lead to the development of methemoglobinemia in infants [6,7], birth defects [8], and colorectal cancers [9]. In aquatic ecosystems, NO_3_-N abundances can create eutrophic conditions, frequently causing fish kills in affected water bodies. Additionally, studies have shown that animals exposed to elevated levels of NO_3_-N may demonstrate endocrine disruption [5]. NO_3_-N is frequently detected in surface, ground, and drinking water due to its frequent use in agricultural and urban landscapes [10,11,12]. In agricultural settings, NO_3_-N is predominantly attributed to the application of commercial fertilizer, animal manure, or municipal biosolids to cropland [9,12].

Some agricultural soil amendments (i.e., manure, biosolids) have been demonstrated to contain other biologically active compounds such as veterinary antibiotics (VAs). Agricultural VAs, particularly those that have been administered to animals for growth promotion or injury treatment and prevention, account for the majority of antibiotics used in many countries [13,14,15]. Consequently, confined animal feeding operations (CAFOs) have increasingly been investigated as hotspots for antibiotic accumulation and potential ecosystem impacts [16,17]. Antibiotics administered in livestock operations have been frequently detected in agricultural soils and have demonstrated the potential to affect nitrogen transformation processes by influencing the population structure of nitrogen-transforming microbial communities and, thus, the activity of N cycle processes, such as nitrification and denitrification in soils, sediments, and adjacent wetland treatment systems [18,19,20,21,22,23,24]. However, differences in the chemical and physical properties of antibiotic compounds impact their bioavailability, mode of action, and degradation routes/rates differently in natural environments. Thus, assessing and/or removing antibiotics from natural systems is challenging [25]. A large concern regarding environmental antibiotic exposures is related to the disruption, elimination, or replacement of microbial community structures in environmental compartments (soil, water, air). Antibiotics rarely occur as a single compound, or even as a singular chemical additive in the environment, adding to the many challenges associated with assessing the impact they may have as mixtures on human and environmental health [26,27,28,29].

Antibiotic deposition and transport (both animal- and plant-administered) is primarily associated with two modes: the direct deposition of animal excreta in and around the feedlot facility and the field application of collected manure–water slurries [29,30]. In both instances, environmental conditions play a key role in determining additional potential transport pathways. In locations that have received manure, runoff and erosion can result in the transport of antibiotics to adjacent surface waters and leachates to groundwater. Plant uptake of antibiotics, both temporary and permanent, presents a removal mechanism from soil and soil interstitial water that can potentially affect transport, moving antibiotics into plant tissue where it is either stored or metabolized [31,32]. Many antibiotics also preferentially adsorb to substrates with high organic carbon contents, such as soils, where the highest concentrations of antibiotics have been detected, which can affect antibiotic transport within an environment [33].

Natural, constructed, and, more recently, floating treatment wetlands (FTWs) are increasingly used across the United States as alternatives to traditional wetlands or water quality treatment systems. Floating treatment wetlands provide an opportunity for unique treatment strategies for dealing with emerging water quality concerns. These act as a well-documented means for mitigating NO_3_-N losses to surface water in regions that are land-limited or have retention ponds. Aside from their nutrient treatment efficiency, floating treatment wetlands are often implemented due to their cost-effectiveness and minimal maintenance requirements. NO_3_-N and VAs are expected to co-occur in agricultural runoff from crop production areas, meaning animal waste lagoons and wetlands used to capture and treat runoff will likely experience the effects of multiple environmental exposures, possibly acting as hotspots for water quality contaminant remediation. 

Although the use of wetlands as a treatment approach for NO_3_-N is well established, the influence that VAs have on N transformation processes in such systems is not yet well understood [34,35,36,37]. Therefore, this study’s overall goal was to assess the cumulative effects of VA residues on nitrogen transformation in wetland environments. This study included three primary objectives: 1. assess VA impact on nitrogen cycle processes in wetland sediments using microcosm incubations, 2. measure the NO_3_-N total mass removal from the water of floating treatment wetland systems over time following the introduction of VA residues, and 3. Identify the fate of Vas in floating treatment wetlands using a mesocosm experiment.

## 2. Materials and Methods

### 2.1. Sample Collection for Microcosms

**Figure 1:**
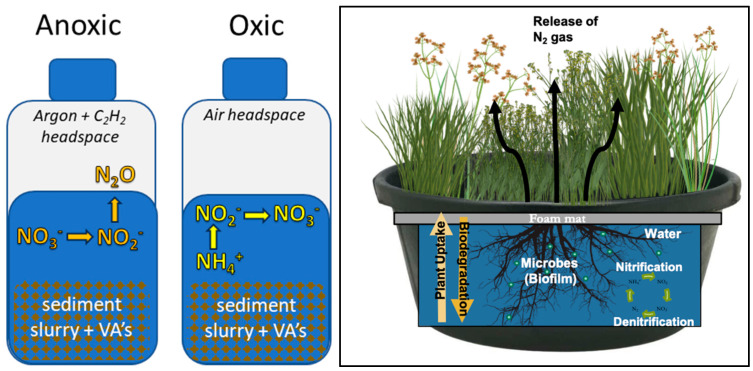
(**Left**) Schematic of anoxic and oxic microcosm sediment plus water slurries, amended with nitrogen and VAs, used for denitrification and nitrification experiments, respectively. Schematic depicts relevant nitrogen processes. (**Right**) Schematic of mesocosm floating constructed wetland, often referred to as a floating treatment wetland (FTW). Once established, plant, soil, and water microbiomes begin to facilitate nitrogen transformation processes. Plant structures are supported by a ~1 in. thick buoyant Beemat and plastic cups to provide structure for root growth.

Microcosm experiments, focusing on the effects of agricultural runoff on nitrification and denitrification rate potentials, were performed using sediment and water collected in December 2019 from the USDA Meat Animal Research Facility (USMARC) near Clay Center, Nebraska (Figure 1). The collection site was the third in a series of lagoons designed to collect runoff from beef cattle feedlots for use in cropland irrigation. The area of the lagoon was approximately 45,000 m^2^, with a depth ranging from 0.5 to 2 m. Wastewater from this lagoon was known to contain VAs [17,29].

Sediment was collected as a grab sample from approximately the top 15 cm of sediment using a shovel and stored in baked, quart-sized glass jars. Additionally, water was collected from the lagoon for chemical characterization. Grab water samples were collected approximately 15 cm below the air/water interface using a 30 mL syringe. Water was filtered through a 0.45 µm capsule filter into 1 L glass bottles. Sediment and filtered water were stored on ice and shipped overnight to the United States Geological Survey (USGS) Laboratory in Boulder, Colorado, within 12 h of collection.

Sediment was homogenized and sieved through a 4 mm sieve and subsamples of the total collected sample were preserved for gravimetric moisture, total carbon (C), and nitrogen (N) content. Sediment KCl-extractable ammonium (NH_4_^+^), nitrate plus nitrite (NO_3_^−^ + NO_2_^−^), and nitrite (NO_2_^−^) concentrations were determined by combining 30 g of wet sediment with 90 g of 2 N KCl and mixing on a shaker table for 2 h. Filtered lagoon water samples were preserved for anions by freezing at −20 °C; cations and total dissolved nitrogen (TDN) by acidification with sulfuric acid and hydrochloric acid, respectively, and dissolved organic carbon (DOC), alkalinity, and pH (Table 1) by chilling at 4 °C.

### 2.2. Microcosm Experimental Setup

Artificial water was prepared based on previously measured water chemistry data from the field site with the exclusion of NO_3_^−^, NO_2_^−^, and NH_4_^+^, which were added separately depending on the assay (Table S1 and Figure S1). Sediment–water slurries were prepared under aerobic and anaerobic conditions, with and without VAs.

The VAs chosen for the mixtures represented four classes. Chlortetracycline (C_22_H_23_CIN_2_O_8_) was chosen from the tetracycline group. Tetracyclines are biologically active against both Gram-positive and Gram-negative bacteria, making them highly effective for use in CAFOs [38]. Monensin (C_36_H_62_O_11_), an ionophore, is used in the cattle and poultry industries as a preventative for coccidiosis. It is also used as a growth promoter in cattle. Lincomycin (C_18_H_34_N_2_O_6_S) is a lincosamide that is used in both veterinary and human medicine (Dungan et al.). In animals, it is typically used in poultry and dairy cattle for the prevention of Gram-positive infections [39]. Sulfadimethoxine (C_12_H_14_N_4_O_4_S) and Sulfamethazine (C_12_H_14_N_4_O_2_S) are used in cattle and poultry to treat a broad range of diseases and are used in both veterinary and human medicine [40,41] (Figure S1). These VAs were selected based on their prevalence in previously completed studies, where each was observed in a significant fraction in wastewater lagoons [29,42]. The artificial water used for the microcosms was amended with none (C0), low (C1), moderate (C2), and high (C3) VA combined concentrations.

#### 2.2.1. Anaerobic Microcosms

Aliquots of the artificial water solutions, with and without VAs, were placed in an anaerobic glove bag overnight to deoxygenate prior to the preparation of the anaerobic denitrification potential microcosm slurry bottles. The following day, slurries were prepared in triplicate by the addition of 12 g of sediment and 100 g of artificial water to glass serum bottles, sealed with butyl rubber stoppers, and then the headspace was flushed with argon gas (Ar). The bottles were then incubated for 3 weeks in the dark using an end-over-end rotator for mixing prior to the initiation of the denitrification potential experiments (Figure S2). 

Following the 3-week exposure period, denitrification potential assays were initiated by flushing the bottles with Ar and then adding an anoxic NO_3_^−^ solution (14 mg N L^−1^, final concentration) to ensure a sufficient nitrogen source over the entire incubation period and 10 mL of acetylene gas (C_2_H_2_) to block the denitrification pathway at the nitrous oxide reduction step. Small-volume (<100 uL) headspace gas samples were removed periodically from the bottles over a 1.5 h time period and analyzed for nitrous oxide (N_2_O) concentrations using a gas chromatograph with an electron capture detector [43] (Figure S2). After the final sampling timepoint, the remaining sediment–water slurry was centrifuged; the supernatant was filtered and preserved for the analysis of cations, anions, and VAs; and the sediment pellet was homogenized and preserved by freezing at −50 °C for DNA analysis. 

#### 2.2.2. Aerobic Microcosms

Air-saturated (oxic) aliquots of the artificial water solutions, with and without VAs, were used for the preparation of the aerobic nitrification potential slurry bottles. The slurries were prepared in triplicate by the addition of 12 g of sediment and 100 g of artificial water to high-density polyethylene (HDPE) screw-cap bottles. Bottles were then mixed on an end-over-end rotator. Background slurries without VAs were immediately sampled by centrifuging the bottle contents, filtering, and preserving the supernatant for anions, cations, and VAs, and then homogenizing and freezing the sediment pellet for DNA. The remaining experimental bottles were incubated for 3 weeks in the dark prior to the initiation of the nitrification potential experiments. After the 3-week period, an oxic solution of NH_4_^+^ (14 mg N L^−1^, final concentration) was added to each bottle to ensure a sufficient nitrogen source over the 48 h period. Slurry samples (5 mL) were removed periodically and then centrifuged, filtered, and preserved for anion and cation analysis. The remaining sediment pellets were preserved by freezing at −50 °C for DNA analysis (Figure S3). 

Samples collected from the VA solutions, background slurries, and the final timepoints for the denitrification and nitrification potential assays were extracted for VAs using hydrophilic–lipophilic-balanced (HLB) binding cartridges and analyzed at the University of Nebraska Water Sciences Laboratory (Lincoln, NE, USA).

### 2.3. Mesocosm Experiment Setup 

The microcosm experiments were critical for establishing the target VA concentrations to assess in the mesocosm experiments as mesocosm treatments were limited due to space limitations. Based on observed nitrogen loss in the microcosm experiments, FTW mesocosms (Figure 1) were enriched with the 1000 µg L^−1^ antibiotic at a University of Nebraska-Lincoln greenhouse facility with ambient climate control to quantify the removal of NO_3_-N and VAs from FTWs. FTW mats (60 cm × 60 cm) were purchased from Beemats (New Smyrna Beach, FL, USA) and planted in 2017 with a mixture of longhair sedge (*Carex comosa*), fox sedge (*Carex vulpinoidea*), swamp milkweed (*Asclepias incarnata*), common rush (*Juncus effusus*), and Torrey’s rush (*Juncus torreyi*). Plants were established two years prior to the experiment. The experiment utilized four triplicate treatments: FTWs without VA enrichments (FTW), FTWs with VA enrichments (FTW-VA), a plant-free aqueous control without VAs (Control), and a plant-free aqueous control with VAs (Control-VA). FTW mesocosms were conducted in black HPDE tanks filled with tap water and did not include sediment and control mesocosms were conducted in black HDPE buckets, both of which have been used in similar mesocosm experiments [44,45,46]. Target concentrations for each VA experimental unit were 1000 µg L^−1^ for each antibiotic and 10 mg L^−1^ KNO_3_ for all experimental units. An Onset HOBO (Bourne, MA, USA) light and temperature sensor was placed in each mesocosm to monitor hourly temperature and light conditions throughout the experiment.

Five days prior to the experiment, all mesocosms were drained and refilled with greenhouse tap water using a flow meter (P3 International Corporation; New York, NY, USA) to approximately 285 L for the experimental tanks and 50 L for lower-volume controls. Water was allowed to sit in the mesocosms for 5 days prior to the experiment to allow any residual chlorine to dissipate. All mesocosms were amended with concentrated KNO_3_ salt solution (Fisher Scientific International, Inc.; Pittsburgh, PA, USA) on the first day of the experiment (Day 0) to achieve initial NO_3_-N concentrations of approximately 10 mg L^−1^. VAs (monensin, sulfamethazine, lincomycin, and chlortetracycline) were dissolved in ethanol (C_2_H_6_O) and then added to the FTW-VA and Control-VA treatments to achieve an initial concentration of 1 mg L^−1^.

Water samples were collected from the mesocosms at 0, 1, 2, 3, 5, 7, and 10 days. Prior to sampling, water in the mesocosms was mixed using a PVC stir rod for 1 min to ensure homogeneity. Water temperature, dissolved oxygen (DO), pH, oxidation-reduction potential (ORP), and conductivity were measured daily using a YSI ProQuatro multiparameter water quality meter (YSI, Yellow Spring, OH, USA). Water samples were collected 15 cm below the surface in 250 mL HDPE bottles, placed in a cooler on ice, and transported immediately to the laboratory, where they were filtered through GF/F filters and stored in a refrigerator until analyzed for NO_3_-N, PO_4_-P, and DOC. VA samples (2 mL) were collected on days 1, 5, and 10 and placed unfiltered directly into amber glass bottles using a 1 mL pipette to be stored frozen until analysis. Water levels within the mesocosms were measured on each sampling day to account for evapotranspiration loss and adjust concentrations.

### 2.4. Analytical Methods

#### 2.4.1. Microcosm Analytical Methods

Water samples were analyzed for major anion and cation concentrations using a Dionex Model ICS-5000 ion chromatograph ([47], Figure S2), total dissolved nitrogen (TDN) using a Skalar Formacs^TN^ Total Nitrogen Analyzer equipped with an ND25 Total Nitrogen detector [48], and DOC using an Oceanographic Instruments Analytical TOC analyzer Model 700 [49]. Nitrous oxide (N_2_O) concentrations were measured using an HNU model 301 gas chromatograph equipped with a ^63^Ni electron capture detector (ECD) (Figure S2). Total C and N content was determined by combustion at 980 °C using an Exeter CE440 Elemental Analyzer [50]. Sediment KCl extracted samples were analyzed for NO_3_^−^ + NO_2_^−^ and NO_2_^−^ concentrations using a Sievers Model 280i Nitric Oxide Analyzer with chemiluminescent detection [51] and for NH_4_^+^ concentration by colorimetric spectrophotometry using the indophenol blue method [52].

#### 2.4.2. Mesocosm Analytical Methods

NO_3_-N and PO_4_-P concentrations from the mesocosm experiment water samples were measured by automated spectrophotometric analysis using a Seal Analytical AQ300 discrete auto-analyzer according to EPA methods 126-D and 134-D, respectively. DOC concentrations were determined using a 1010 TOC Analyzer (Oceanography International Corporation; College Station, TX, USA) with Standard Method 5301D.

#### 2.4.3. Microbial Analytical Methods

Nucleic acids were extracted from sediment samples collected during the microcosm experiments using the DNeasy PowerSoil kit following manufacturer protocols (Qiagen, Inc., Germantown, MD, USA, document #HB-2266-002), stored at −20 °C, and then sent to Michigan State University’s Research Technology Support Facility for Next-Generation Sequencing. Illumina amplicon libraries and iTags (Illumina, Inc., San Diego, CA, USA) were generated by amplification of the V4 hypervariable region of bacterial and archaeal 16S rRNA genes using dual indexed, Illumina compatible primers (515f/806r) [53] and sequenced following a standard protocol [54]. Additional details of the sequencing methods and data can be found in Repert et al. (2024) [55].

#### 2.4.4. Antibiotic Analytical Methods

VAs and degradation products were quantified using solid-phase extraction coupled to liquid chromatography–tandem mass spectrometry (LC-MS/MS) on a Waters Quattro Micro or Agilent 6410 triple quadrupole mass spectrometry system for water, sediment, and biomass samples [56,57,58,59]. Stable isotope analogs were used for quantification when available and surrogates were used to monitor analyte recovery in each method [56].

In summary, samples were processed and analyzed for antibiotics using modifications of several previously published methods [56,57,58,59]. Calibration standards (Sigma Aldrich, St. Louis, MO, USA) and reagents (Thermofisher Scientific Acros, St. Louis, MO, USA) were used in the analysis to ensure accuracy of results. Labeled sulfamethazine phenyl-13C6 was purchased from Cambridge Isotope Laboratories (Andover, MA, USA). Calibration standards and spiking solutions were prepared in methanol (Optima grade, Thermofisher Scientific, St. Louis, MO, USA). Frozen plant tissue samples were divided, coarsely ground with a homogenizer, and 0.2 g portions were weighed out into 50 mL centrifuge tubes. Each sample was spiked with 100 ng of demeclocycline and sulfachloropyridazine surrogates, mixed with 2 mL of McIlavine-EDTA buffer (pH = 5) plus 5 mL of acetonitrile, and shaken for 20 min on a wrist action shaker. After centrifuging at 3000 rpm for 10 min, the solvent extract was decanted into a 100 mL Rapid Vap (LabConco Corporation, Kansas City, MO, USA) evaporation tube. Samples were mixed with another 5 mL acetonitrile, vortexed for 30 s, centrifuged, and combined with the first extract. Plant extracts were concentrated under nitrogen at 35 °C to approximately 1 mL and mixed with an additional 50 mL of McIlavine-EDTA buffer. The aqueous extract was passed through a preconditioned 200 mg HLB polymeric solid-phase extraction cartridge (Waters Corporation, Milford, MA, USA), followed by elution with 10 mL of acetonitrile and 10 mL of 0.1% ammonium formate (pH = 5) in methanol. After concentrating under N_2_ gas to approximately 500 µL, extracts were mixed with 100 µL of 1 ng µL^−1^ internal standard spike solution (100 ng sulfamethazine-phenyl-13C8 and doxycycline) and further concentrated to 100 µL, mixed with 300 µL of high-purity reagent water, and transferred to a silane-treated insert contained in an autosampler vial. Aqueous samples (80 µL) were measured out into an autosampler vial insert, mixed with 100 µL of surrogates, 100 µL internal standards, and 520 µL high-purity reagent water and mixed to produce an 80:20 water–methanol mix. 

Extracts and aqueous samples were analyzed on a Quattro Micro triple quadrupole mass spectrometer interfaced with a 2695 high-pressure liquid chromatography system (Waters Corporation, Milford, MA, USA). A Thermo HyPURITY C18 column (250 mm × 2.1 mm ID, 5 µm particle size) at 50 °C and a flow rate of 0.20 mL/min was used for separation with a gradient mix of (A) 1 mM citric acid in methanol and (B) 1 mM citric acid in water. Initial conditions were 5%A, which was immediately increased to 20%A, then to 30%A at 2 min and 95%A at 5 min, and then held until 18 min. The column was flushed with 10% formic acid in methanol before switching back to initial conditions (5%A) at 24 min. The column was re-equilibrated for 11 min before the next injection. Tandem mass spectrometry in positive ion mode (ESI+) used the pseudo-molecular ion [M + H]^+^ or adduct ion for fragmentation for monensin, and corresponding fragment ions were selected for identification and quantitation. Ionization and collision energies were optimized based on procedures described by the instrument manufacturer. The collision gas was argon at 4.0 × 10^−3^ torr, nitrogen desolvation gas flow was 600 L h^−1^, and cone gas was 30 L h^−1^. The ESI+ source temperature was operated at 120 °C with a capillary voltage of 4 kV. The cone voltages and collision energies used for each standard and analyte are given in Table 2. Sulfamethazine-phenyl-13C8 was used as the internal standard for all analytes except chlortetracycline, which used doxycycline. Instrument detection limits, determined as 3 times the standard deviation of the lowest standard (2.5 µg L^−1^), ranged from 0.16 for sulfamethazine to 3.3 µg L^−1^ for chlortetracycline. Method detection limits, determined from 8 replicates of a clean sand matrix (1 g fortified at 1 ng g^−1^), ranged from 0.23 ng g^−1^ for lincomycin to 1.8 ng g^−1^ for tylosin. Quality controls included analysis of laboratory reagent blanks, laboratory fortified blanks, fortified matrices, and duplicates at a frequency of 5% each.

### 2.5. NO_3_-N Removal Rates

Changes in NO_3_-N concentrations in the mesocosm experiments were fit to a first-order decay response model [44,60,61,62,63]. First-order removal rate constants (*k*) were determined for each treatment in the mesocosm experiments using the following equation:

$$k=-\left(\frac{ln\frac{C_{i}}{C_{t}}}{t}\right)$$

where *C_i_* was the initial NO_3_-N concentration (mg L^−1^), *C_t_* was the final NO_3_-N concentration at the end of the experiment (mg L^−1^), t was the experiment time (hour), and *k* was the removal rate constant (hour^−1^). Aerial NO_3_-N removal rates were determined for each treatment using the following equation [62]:

$$J_{NN}=\frac{{(N}_{Applied}-N_{Remaining})}{A*t}$$

where *J_NN_* was the NO_3_-N removal rate (mg m^−2^ day^−1^), *N_applied_* was the NO_3_-N load from the enrichment (mg), *N_Remaining_* was the NO_3_-N remaining in the wetland mesocosm on the sampling day (mg), A was the surface area of the treatment wetland mat (m^2^), and *t* was time following nutrient enrichment (day).

### 2.6. Statistical Analyses

Significant differences were assessed using Minitab 17 (Champaign, IL, USA, 2020), with a reported significance of α = 0.05. Nutrient data were evaluated for normality and, where necessary, transformed using a log transformation to better fit a Gaussian distribution. An analysis of variance (ANOVA) was conducted on the mean NO_3_-N concentrations by treatment. In addition, a Tukey’s post-hoc Honest Significant Difference (HSD) comparison was conducted to compare significant differences in treatments by sampling day in the mesocosm experiment.

Data for VA concentrations in water and plant tissue were evaluated for normality and, if necessary, normalized by log transformation to better fit a Gaussian distribution. A *t*-test was conducted on the above- and below-surface biomass VA concentrations after a one-way ANOVA revealed significant differences in the data. 

## 3. Results

### 3.1. Microcosm Experiments

#### 3.1.1. Microcosm Experimental Conditions

Concentrations of chlortetracycline, lincomycin, monensin, and sulfadimethoxine in the VA mixture stock solutions (C0, C1, C2, and C3) added to the microcosm experimental bottles ranged from <0.008 to 35.5, 0.16 to 1682, <0.033 to 324, and <0.013 to 631 µg L^−1^, respectively (Table 3). Background mean concentrations of the antibiotics in the preincubation, no-VA-addition slurries were 0.559, 0.031, 0.133, and 0.119 µg L^−1^ for chlortetracycline, lincomycin, monensin, and sulfadimethoxine, respectively (Table 3 and Table S2). Samples collected at the end of the nitrification and denitrification experimental incubation periods were lower in all antibiotic concentrations for all treatments compared to starting stock concentrations (Table 3 and Table S2). 

#### 3.1.2. Nitrification Potential Microcosm Experiments

**Figure 2:**
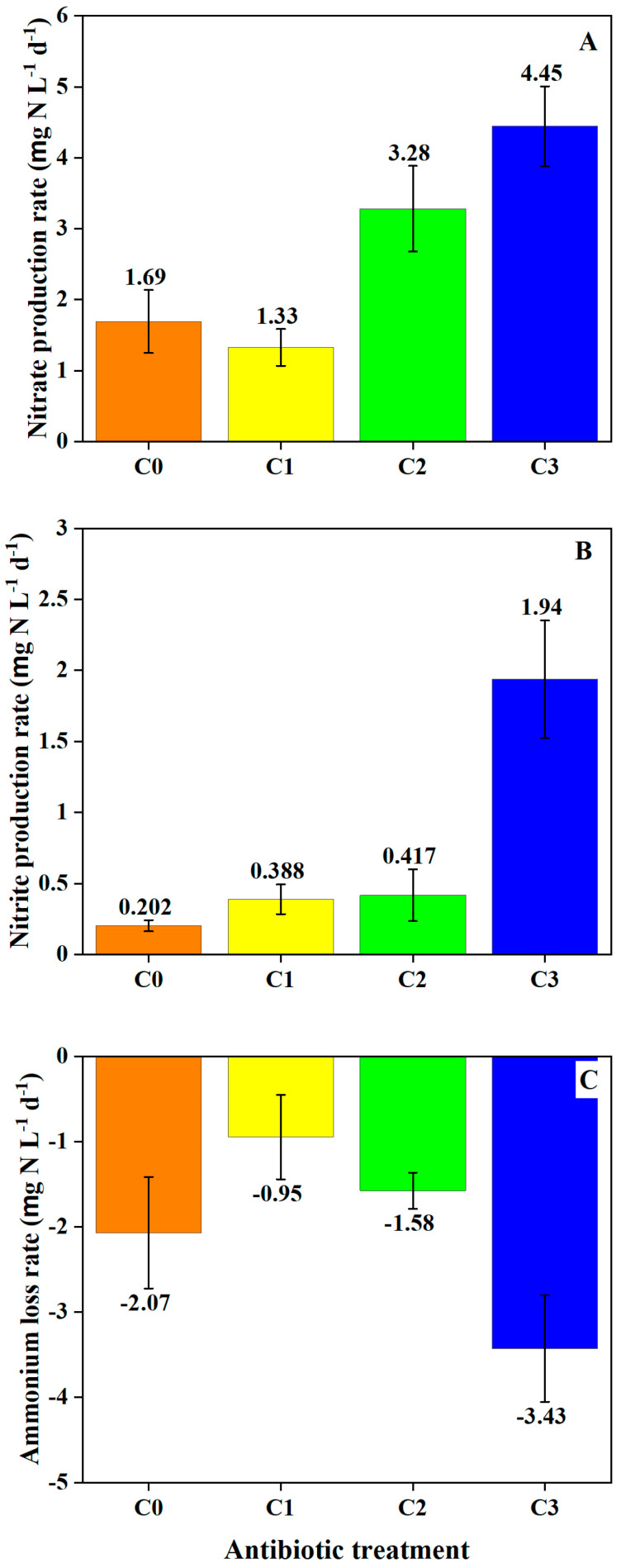
Rates of potential (**A**) NO_3_^−^ production, (**B**) NO_2_^−^ production, and (**C**) NH_4_^+^ removal over time in oxic sediment plus artificial pond water slurries with NH_4_^+^ and VA. Error bars represent standard error of the slope of the concentration change with time.

Nitrification was evidenced in oxic sediment–water slurries by NO_3_^−^ and NO_2_^−^ production rates from added NH_4_^+^ (Figure 2 and Figure S4). Rates of NO_3_^−^ production were significantly greater in the C3 VA mixture treatment compared to the C0 control and C1 treatment (Figure 2; *p*-values < 0.005). NO_2_^−^ production similarly increased with increasing VA concentration, exhibiting rates significantly greater in the C3 treatment compared to all other treatments (*p*-values < 0.0001). NH_4_^+^ removal rates were likewise greater in the C3 VA treatment (*p*-values < 0.05), with no obvious differences between the C0, C1, and C2 treatments.

#### 3.1.3. Denitrification Potential Microcosm Experiments

**Figure 3:**
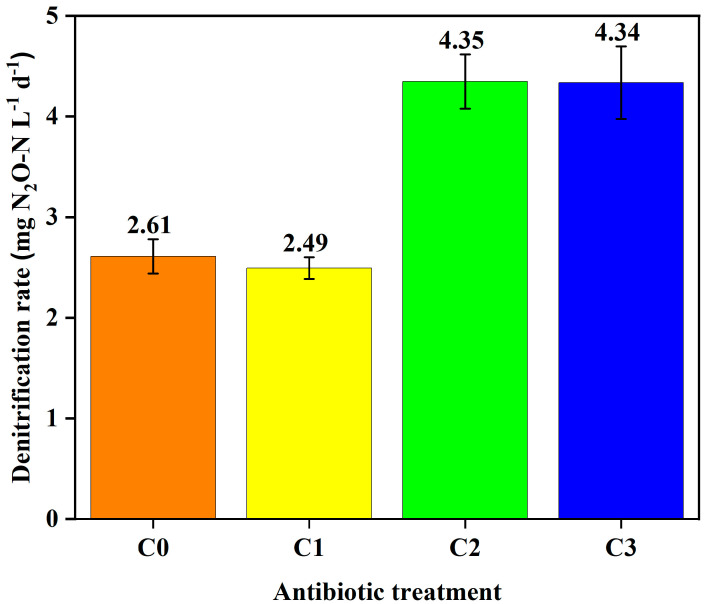
Rate of denitrification potential (as N_2_O production over time in the presence of acetylene) in sediment plus artificial pond water slurries with NO_3_^−^ and VA. Error bars represent standard error of the slope of the concentration change with time.

Denitrification potential rates were not inhibited by the VA mixture in all treatment doses (Figure 3). Instead, rates of N_2_O production were stimulated in the presence of VAs, especially at the two higher concentrations (C2, C3; *p*-values < 0.05) (Figure 3).

**Figure 4:**
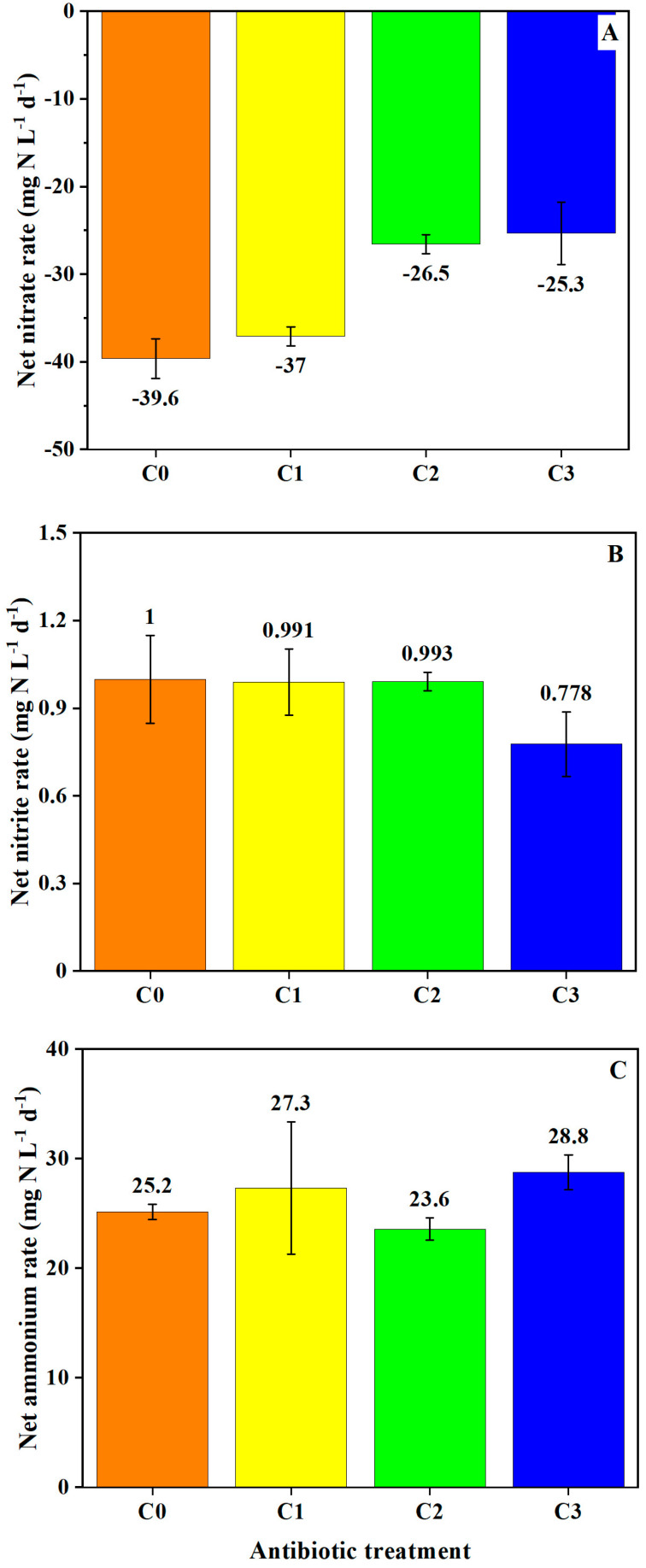
Estimated rates of (**A**) NO_3_^−^, (**B**) NO_2_^−^, and (**C**) NH_4_^+^ concentration changes over time in denitrifying sediments slurried with artificial pond water containing VA addition. Estimated starting concentrations and measured final concentrations in the sample bottles were used to calculate potential rates. The rate and corresponding error bars represent the average and standard deviation of three values.

Estimated rates of NO_3_^−^, NO_2_^−^, and NH_4_^+^ production or loss in the anoxic sediment–water microcosms were determined by the differences in water samples collected from initial background sediment–water slurries and water samples collected from the denitrifying sediment slurries at the end of the incubation period. Initial NO_3_^−^ concentrations included the estimated concentration of 14 mg N L^−1^ added for the denitrification assay. NO_3_^−^ loss was greater in the C0 and C1 VA incubations, while NO_2_^−^ production was greater in the C0, C1, and C2 VA incubations (Figure S5). However, overall rates of production or loss of NO_3_^−^, NO_2_^−^, and NH_4_^+^ were not significantly different between VA concentrations (Figure 4).

#### 3.1.4. Microbial Community Variability 

**Figure 5:**
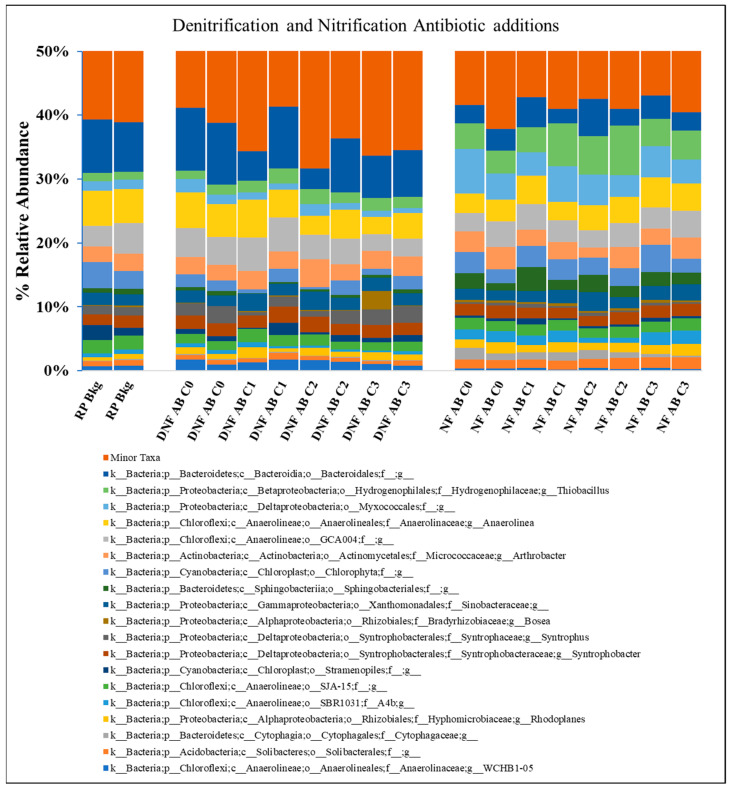
Stacked bar plot showing percentage (%) of relative abundance of dominant (>1.5% maximum abundance) genus-level classification based on 16S rRNA sequencing from reuse pit (RP) background (Bkg) sediments collected 17 December 2019 and final timepoints from denitrification (DNF) and nitrification (NF) slurried sediment samples collected 8 January 2020 and 9 January 2020, where AB = antibiotic. The final concentrations for the respective antibiotics for C0, C1, C2, and C3 are shown in Table 3. Duplicates shown in the figure for RP Bkg were collected from the original sieved and homogenized sediment. Duplicates shown in the figure for the DNF and NF experiments were collected from replicate incubation bottles 1 and 2.

**Figure 6:**
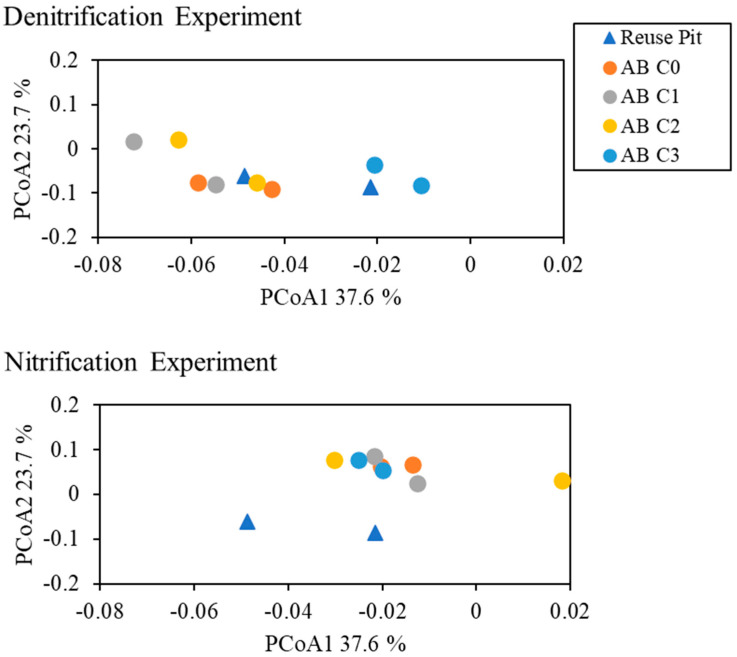
Principal component analysis of weighted UniFrac distances showing relative relatedness of microbial community members in the denitrification and nitrification experiments amended with the antibiotic (AB) mixture and relative to the background reuse pit sample. The final concentrations for the respective antibiotics for C0, C1, C2, and C3 are shown in Table 3. Duplicates shown in the figure for RP Bkg were collected from the original sieved and homogenized sediment. Duplicates shown in the figure for the DNF and NF experiments were collected from replicate incubation bottles 1 and 2.

Overall, the greatest difference in the microbial communities was between the anoxic denitrification and oxic nitrification samples collected at the end of the month-long incubation and experimental period, rather than between the different VA amendments for the two experiment types (Figure 5). The microbial communities in the reuse pit (RP) background sample and the denitrification (DNF) incubations were dominated (>4%) by members of the Bacteriodales and GCA004 orders and the *Anaerolinea* genus from the order Anaerolineales. The dominant microbial community members in the nitrification incubations came from the Bacteriodales and Myxococcales orders and the Thiobacillus genus from the order Hydrogenophilales. A principal component analysis (PCoA) indicated relatively little difference between the microbial communities in the denitrification incubations and the original reuse pit microbial community, but some difference between the lower antibiotic treatment concentrations (C0 to C2) and the higher concentration (C3) (Figure 6). There was little difference in the microbial communities in the nitrification incubations at the different VA concentrations, but greater divergence from the original reuse pit sediment. Additionally, there was an evident difference when comparing the denitrification communities with the nitrification communities, with greater dissimilarity between these two groups as evidenced by their position on the PCoA 1 axis.

### 3.2. Mesocosm Experiment

#### 3.2.1. NO_3_-N Removal following Exposure to Veterinary Antibiotics

**Figure 7:**
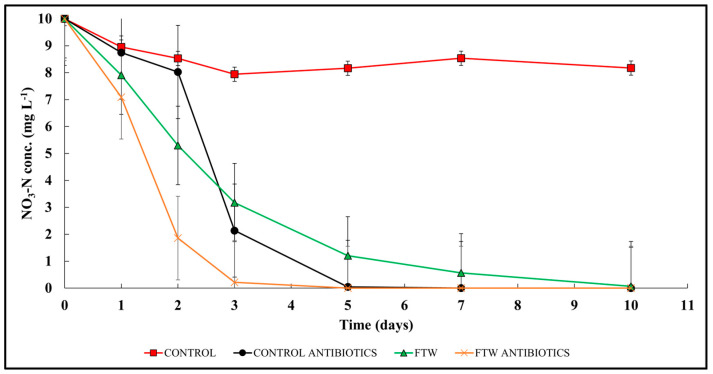
NO_3_-N removal in the mesocosm following NO_3_-N and VA enrichment. Significant differences between treatments were evaluated with Tukey mean comparison.

Mesocosms were constructed with floating treatment wetlands (FTWs) to test NO_3_-N removal following exposure to VAs. The VA concentrations used were based on significant findings from the microcosm experiments. After exposure, NO_3_-N concentrations significantly decreased with time in all treatments except the water-only Control (*p*-value = 0.002; Figure 7). Significant differences were observed between the FTW, FTW-VA, and Control-VA treatments on day 2, but were insignificant for the remainder of the study after the NO_3_-N concentration was >75% depleted in each treatment. Average NO_3_-N removal rates were significantly higher in the FTW and Control-VA treatments compared to the water-only Control (*p*-value = 0.002, Figure 7). Average NO_3_-N removal rates were 50 ± 81 mg m^−2^ day^−1^ in the water-only Control (with a range of −70 to 203 mg m^−2^ day^−1^ dependent on the day of the study), 658 ± 602 mg m^−2^ day^−1^ in the Control-VA (with a range of 130 to 1595 mg m^−2^ day^−1^ dependent on the day of the study), 1508 ± 806 mg m^−2^ day^−1^ in the FTW (with a range of 368 to 2781 mg m^−2^ day^−1^ dependent on the day of the study), and 2759 ± 1084 mg m^−2^ day^−1^ in the FTW-VA (with a range of 1800 to 4618 mg m^−2^ day^−1^ dependent on the day of the study).

#### 3.2.2. Physiochemical Fluctuations

Physiochemical concentrations varied throughout the mesocosm experiment (Table 4). The DO ranged between 0.1 and 7.6 mg L^−1^ in the water-only Control, 0.1 and 6.7 mg L^−1^ in Control-VA, 0.3 and 1.3 mg L^−1^ in FTW, and <0.1 and 1.6 mg L^−1^ in FTW-VA treatments. Average water temperature ranged from 25.5 to 27.6 °C in the mesocosms, with Control and Control-VA treatments being significantly warmer than the FTW and FTW-VA treatments due to shading created by the mats and the larger volume of water in the FTW treatments (*p*-value < 0.001). No significant differences were observed in pH between treatments (*p*-value = 0.470), where pH values ranged between 6.8 and 7.0. Average DOC concentrations throughout the experiment were significantly higher in the treatments that received VAs compared to the treatments that did not receive VAs (*p*-value < 0.001), with DOC concentrations ranging between 2.9 and 6.0 mg L^−1^ in the water-only Control, between 10.2 and 16.7 mg L^−1^ in FTW, between 56.7 and 104 mg L^−1^ in Control-VA, and between 62.8 and 89.6 mg L^−1^ in FTW-VA. The wide range of DOC values was due to the use of ethanol to dissolve the VAs prior to enriching the mesocosms. Approximately 170 mg L^−1^ (98.7% of the total) of C was added as an ethanol solvent. The addition of C from VAs accounted for 3.7 mg L^−1^ (1.3% of the total) of the C added in contaminant spikes. While, preferably, the DOC would have been consistent between the treatments, NO_3_-N removal was not found to be limited following the addition of the VAs but was instead potentially enhanced by the addition of ethanol. Except for the water-only Control treatment, all other mesocosms had parameters suitable to sustain and enhance denitrification [64].

#### 3.2.3. VA Recovery in Water

VAs in the mesocosm water were sampled on days 1, 5, and 10. The mass recovered ranged from below detection limits to 364.5 mg (Table 5). VA concentration generally increased with sampling day, with day 10 averaging the highest mass recovery out of all sampling days (Table S4). Chlortetracycline had little to no recovery on any sampling day, while lincomycin had the largest recovery each day and on average over the 10-day period. Day 10 recoveries for lincomycin, monensin, and sulfamethazine were near or slightly exceeded the target spike concentration (1 mg L^−1^), while chlortetracycline was not recovered at a substantial fraction in the water (Table S4). All VA concentrations in treatments not administered VAs (Control, FTW) were below detection limits on all sampling days. 

#### 3.2.4. Physiochemical Relationships to Antibiotic Concentrations

Antibiotic concentrations measured on days 1, 5, and 10 of the experiments varied between treatments (Table 5; Figures S6–S9). Monensin concentrations increased in the Control-VA while lincomycin, monensin, and sulfamethazine concentrations increased in the FTW-VA treatments during the experiment. Given these concentration changes, dissolved oxygen, ORP, pH, conductivity, water temperature, and DOC were also assessed in tangent with the antibiotics to assess the potential for release from absorption to organic matter during the experiment. Overall correlations were weak and inconclusive. Physiochemical relationships with R^2^ > 0.5 were notable. Chlortetracycline was found to increase with increasing ORP for the FTW-VA, while lincomycin was observed to increase with increasing water temperature for the C-VA. Monensin was observed to decrease with increasing dissolved oxygen and DOC for the CA-VA. Sulfamethazine was observed to increase with increasing concentrations of dissolved oxygen, ORP, pH, and DOC, while concentrations decreased with increasing water temperature for the C-VA treatment.

#### 3.2.5. Plant Uptake of Veterinary Antibiotics

**Figure 8:**
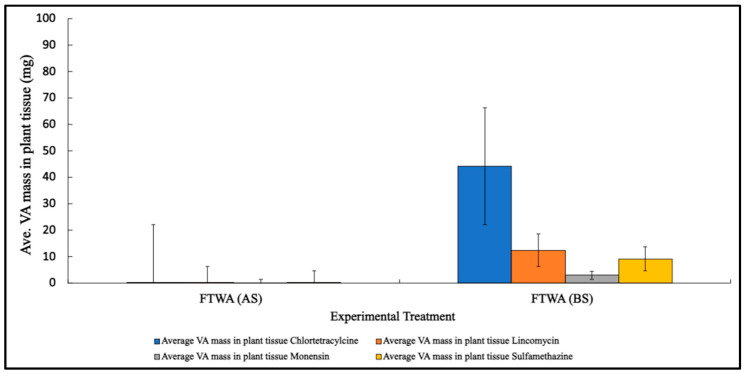
VA concentrations in plant biomass samples taken below (BS) and above (AS) the FTW mat surface. Trace levels of VAs were observed in the AS samples, while substantial amounts were recovered for quantitative analysis in the BS sampling area. Standard deviation bars for each VA constituent were reported.

The uptake and/or sorption of VAs to plant tissue was evaluated by separating and analyzing the above- and below-surface biomass samples from the FTWs. Each of the four VAs added to FTW-VA treatments was detected during analysis, with chlortetracycline being the main constituent recovered (17.37% of the initial spike). Above-water-surface (AS) plant tissue VA concentrations recovered were minimal, with only 0.9 mg m^−2^ in total across all VAs. Instead, VAs were observed to accumulate primarily in the below-water-surface (BS) of the mat plant tissue. A total of approximately 191 mg m^−2^ was recovered across all four VAs assessed, with chlortetracycline as the primary contributor (122.8 mg m^−2^) (Figure 8). Concentrations were representative of accumulation over the 10-day mesocosm experiment. To assess significant differences within and between treatments, data were log-transformed to achieve normality, and a two-way ANOVA without replication was completed using treatment averages of areal concentrations for VAs in plant biomass (Table S3). Significant differences between the sampling locations (BS vs. AS) and/or treatment types were observed (*p*-value = 0.05), and two additional one-way ANOVA tests indicated that both collection location (*p*-value = 0.02) and treatment type (FTW vs. FTW-VA) (*p*-value = 0.01) were significant factors in the accumulation and detection of VAs. However, no significant differences were observed between the four VA types assessed (*p*-value = 0.77).

## 4. Discussion

The current literature surrounding VA impacts on human and environmental health focuses largely on single compound testing to assess the impact of VA class/species and physiochemical behavior in various environments [65,66,67]. In many of those studies, several compounds were examined, but not as mixtures with one another. While the need for comparison studies to determine compound-specific effects is crucial to understanding the interactions of a mixture, the literature surrounding the impact of mixtures on an ecosystem or in a controlled environment is lacking. In both parts of our study, we were unable to associate an effect with a single antibiotic compound. Rather, any impact represented the aggregate, net effect of the mixture, with the results indicating that there may have been complex, differential effects of the individual VA compounds within the mixture on nitrification and denitrification rates.

### 4.1. Microcosm Nitrification Rates

In our microcosm study, the addition of VAs appeared to stimulate nitrification rates at higher VA concentrations. NO_2_^−^ and NO_3_^−^ produced in our experiments totaled 6.39 mg N L^−1^ d^−1^ at the C3 VA treatment. This finding was not consistent across the existing literature, with several studies reporting inhibition, others reporting no impact, and some reporting stimulation of nitrification rates when exposed to various VAs. A 2016 review investigating the impacts of antibiotics on the terrestrial N cycle examined the impact of 18 VA compounds at concentrations ranging from 0.0003 to 500 mg kg^−1^, most as single compound assessments [68], and found varying results. The studies varied in duration, spike concentration, assessment type (single/multi), and media. Three of the antibiotic classes and species assessed in the review for impacted nitrification rates were also tested in our study, (ionophore–monensin, sulfonamide–sulfadimethoxine, tetracycline–chlortetracycline).

Monensin was evaluated as a mixture at three different concentrations (0.01–0.1 μg L^−1^, 0.01–10 μg L^−1^, 100 μg L^−1^) over similar time intervals to our study. Monensin has previously had no effect on nitrification potential rates [69,70]. Sulfadimethoxine was evaluated in the same study as monensin [70] but inhibited nitrification rates in soil microcosms spiked at 200 mg kg^−1^. Chlortetracycline was investigated in soil microcosms from 0.0003 to 0.03 μg L^−1^ over a two-week evaluation period, where chlortetracycline had no effect on nitrification [71]. Similarly, Katipoglu-Yazan et al. (2013) reported no change in nitrification results with increasing chlortetracycline concentrations. Findings for all three VAs varied by compound, further demonstrating the complexity of assessing VA mixtures. Findings from this and other studies suggest that nitrification rates may vary substantially based on the study design and environmental conditions and that concentrations of VAs may be a significant factor in the observable effects of VAs on wetland microbial processes.

### 4.2. Microcosm Denitrification Rates

In our microcosm study, the addition of VAs appeared to stimulate denitrification rates at VA C2 and C3 treatments. Denitrification was measured at 4.35 and 4.34 mg N_2_O L^−1^ d^−1^ for the two treatments, suggesting a potential impact of VA concentration on the stimulation of denitrification when in the presence of VA mixtures.

Three of the antibiotic classes were assessed in a 2016 review [68] for their impact on denitrification rates. The studies included three antibiotic classes evaluated in our study (ionophore, sulfonamide, tetracycline) and two of the same VAs (sulfamethazine, chlortetracycline). A study evaluating an ionophore (narasin) reported stimulated denitrification from one to four days when exposed to a concentration of 0.000001–0.001 mg L^−1^, but subsequently inhibited denitrification after more than five days [72]. Chlortetracycline was evaluated in groundwater from 0.01 to 1 mg L^−1^ and was observed to inhibit denitrification over the seven-day experiment, with higher inhibition rates at higher spike concentrations (Ahmad et al., 2014). Sulfamethazine was evaluated in both sediment and groundwater for a period of hours to days, showing inhibitory effects at both low (0.00005–0.100 mg L^−1^) and high (0.01–1 mg L^−1^) concentrations [68,73,74]. Lincomycin and other lincosamides were not assessed in the 2016 review; however, additional investigations of the effects of lincosamides, including lincomycin, have noted an inhibitory effect of the lincosamides on nitrogen transformation, specifically in that of anaerobic bacteria [75]. Findings from these studies are mostly inconsistent with the findings from our microcosm study, as the inhibition of denitrification was not observed at any assessed concentration in our microcosms. These results suggest that, like the nitrification results, denitrification rates may vary substantially based on the study design and environmental conditions, and VA concentrations may be a significant factor in the observable effects of VAs on wetland microbial processes. It is also possible that experiment duration plays an important role as studies have reported that inhibition and stimulation impacts may vary based on VA exposure duration [72].

### 4.3. Microcosm Microbial Community 

In this study, the microbial communities in the microcosms were exposed to a VA mixture 3 weeks prior to the commencement of the experiments. There were no great changes in microbial community composition; however, some variation was apparent. The microbial community composition of the anaerobic denitrification microcosms was noticeably different from the aerobic nitrification microcosms, but similar to the original RP sediment (Figure 5 and Figure 6). This suggests that the anaerobic microbial communities may have already been adapted to incubation conditions and the VA mixtures used in this study. The sediment in the lagoon was likely anoxic year-round, such that denitrification might be expected to be a predominant process and that the microbial community structure in the anaerobic microcosms would be typical of what was found in that environment. While community members of the Bacteroidales and Anaerolineales orders were found in both the anaerobic and aerobic experimental conditions, these orders were generally more abundant in the denitrification experiments and the original RP sediment. These orders include known denitrifiers [73] and have been shown to dominate the microbial community in anaerobic digesters containing pharmaceutical wastewater [74]. In addition, there was an increase in the relative abundance of minor taxa with increasing concentrations in the denitrification microcosms (Figure 5), which could be related to taxa with the ability to anaerobically degrade the VA mixture [75].

Antibiotics can have significant effects on microbial biodiversity and ecological functioning, leading to changes in biogeochemical processes including nitrogen transformation [66,76,77,78,79,80]. In this study, both nitrification and denitrification rates increased with higher VA dose concentrations (Figure 2 and Figure 3). The PCoA analysis demonstrated a divergence of the C3 denitrification microbial community from the C0, C1, and C2 communities, but little difference from the original RP sediment. The combined effect of antibiotics in aquatic environments can lead to significant effects on aquatic organisms even when the concentration of the individual antibiotics has no effect [78,81]. In addition, highly sorptive antibiotics, such as tetracyclines, as well as the toxicity of the VA degradation products, could have significant effects on sediment microbial [82,83]. 

### 4.4. Mesocosm Physiochemical Parameters

NO_3_-N removal in wetlands is typically dominated by a combination of two NO_3_-N removal processes: denitrification at the water/soil/plant interface and plant uptake. While the microcosm experiments assessed microbial processes, mesocosm experiments added in the factor of plant contributions. In the mesocosm study, parameters were assessed to ensure optimal conditions for denitrification to occur in the treatment tanks. Some of those parameters included DO (<0.35 mg L^−1^), water temperature (>0 °C), pH (<6.5–7.5), and DOC [64,84,85]. This was different from the microcosm experiment as laboratory conditions were specifically set up to perform various controlled nitrogen transformations. In the mesocosm experiment, all treatments met the criteria considered to be required for denitrification, aside from the DOC value in the Control treatment. This was likely due to the addition of ethanol as the VA mixture solvent, which may have impacted denitrifying bacteria by providing a carbon source, creating optimal conditions for denitrifying microbial communities [64,86,87]. The Control treatment did not receive any VAs and had substantially lower DOC than those that did.

### 4.5. Mesocosm Nitrate Reduction

Results from the nitrate reduction mesocosm study paralleled the microcosm results for denitrification. The FTW-VA (*k* = −1.28) treatment demonstrated the fastest reduction of NO_3_-N, followed by Control-VA (*k* = −1.07), FTW (−0.50), and Control (*k* = −0.02). The use of floating treatment wetlands as potential systems for emerging contaminant treatment is a relatively new concept, and, thus, few studies have assessed the implications of agrochemical introduction to wetland ecosystems and how that may impact nutrient cycling and other environmental health factors. Lindgren et al. (2022) evaluated neonicotinoids in constructed floating treatment wetlands, reporting that the pesticide did not significantly affect nitrate reduction in floating wetlands [60]. This result is again consistent with the results from both experiments in our study that little to no influence is observed by agrochemicals until they are observed at elevated levels.

To elucidate why the Control-VA treatment reduced nitrate-N at such a substantial rate, we initially looked at the dissolved organic carbon differences in the tanks/buckets. DOC even in a water-only treatment may have been enough to provide carbon to a denitrifying bacterial community. However, several factors may have affected the experiment in a way that caused a significant reduction in the Control-VA tank, namely, algae production. Algae growth, despite efforts to prevent and remove it from controlled experiments, is a challenge all wetland mesocosm studies encounter. Algae were not sampled in this study for nitrogen content or VAs, but other greenhouse mesocosm studies have reported algae growth in tanks that were designated water-only controls and suggested that some of the nitrate-N reduction occurring throughout the experiment was driven by algae consumption [44].

### 4.6. Mesocosm Plant Biomass VA Uptake/Adsorption

The majority of plant uptake observed in this study was chlortetracycline accumulation in the root biomass. In total, 44.2 mg of chlortetracycline was recovered on average from the below-surface biomass, while none of the other three antibiotics had any more than moderate accumulation. The current literature reports similar trends in bioaccumulation, specifically in edible plants receiving manure amendments containing trace amounts of VAs [88,89]. Notably, Zhou et al. (2024) also reported the effectiveness of phytoremediators to remove sulfonamides from aquatic environments [90]. The study did not report a significant inhibition of nitrogen-transforming microbial communities and instead concluded that any potential inhibition of the bacterial communities by VAs in the study may have been reduced or obscured due to the presence of the plants and their root exudate expression. In these previous studies, VAs were observed to be taken up into different parts of the plant, including the leaves, stems, and root zone. High concentrations of chlortetracycline in plant roots have also been observed in some edible plants, with the observed inhibition of cell division highlighting one of the potential ecotoxicological concerns that VAs may produce. However, this impact, like many of the impacts observed by VAs, was inconsistently observed across various VA concentrations [91,92,93].

### 4.7. Agrochemical Mixture Studies

Of the mixture studies reviewed, only one observed an inhibitory response to nitrogen transformation. Xu et al. (2020) evaluated sulfamethoxazole (SMX) with 2-Ethylhexyl-4-Methoxycinnamate, a sunscreen byproduct, and found that both inhibited microbial denitrification gene expression, thus inhibiting denitrification [94]. Conversely, several recent studies have reported insignificant differences in nitrogen transformation between VA and non-VA treatments similar to our mesocosm experiment. Gray and Bernhardt (2022) evaluated the difference between VA impact as single compounds and as mixtures and found no significant difference in nitrogen transformation between treatments with and without VAs [95]. Dang et al. (2021) measured the rates of nitrogen transformation by metagenome sequencing from a reservoir known to be contaminated with VAs and similarly reported that no significant impact on nitrogen transformation was observed [96]. The results from these studies continue to highlight the conflicting information being reported when assessing both single and multi-compound mixtures, especially in natural or non-controlled conditions.

## 5. Conclusions/Future Work

This study conducted a multi-scale coupled microcosm-mesocosm experiment to investigate the implications of VAs entering wetland environments. In the microcosm sediment slurry experiments, observed nitrification and denitrification rates for the VA treatments appeared largely dependent upon the concentrations of VAs that were added to each microcosm. In the microcosm experiment, low VA concentrations (<15 μg L^−1^, final aqueous concentration) did not impact nitrification and denitrification potential rates. However, high VA concentrations (>50 μg L^−1^, final aqueous concentration) resulted in increased nitrification and denitrification rates. Not all findings were consistent with that of other current literature, suggesting that there remains a significant amount of uncertainty in the impact of VA mixtures on wetland ecosystems.

The mesocosm study mirrored conclusions from the microcosm study in that nitrate reduction was not inhibited by adding a high concentration of the VA mixture, but instead resulted in a stimulatory effect on NO_3_-N reduction. Several possibilities exist as to why the addition of VAs stimulated NO_3_-N reduction in both FTW and Control treatments. We believe that it was likely a combination of the growth and nutrient consumption of algae in the experimental tank and/or the addition of ethanol as the solvent for VA administration into the experimental tanks. Each of these could have accounted for the significant increase in NO_3_-N reduction early in the experiment. Interestingly, plant absorption of the VAs, specifically chlortetracycline, was almost exclusively limited to the roots. The remaining three VAs were almost exclusively recovered from the water. These findings provide new insight into the fate of VAs in agroecosystems and downstream wetland vegetation. Further, fate and transport observations like these allow for more informed decision-making processes in the management and implementation of FTWs receiving agrochemical mixtures for water quality treatment. Further research is needed to determine by which mechanism VAs impact denitrification processes at larger scales.

To better describe how VA interactions occur, future work may implement continuous measurement probes to sample at a higher frequency for each constituent. Future experiments and studies should also consider employing high-resolution, long-term datasets to better understand VA interactions with wetlands and how VA residence time plays a role in the alteration of bacterial communities, nutrient cycling, and the development of antibiotic-resistant genes.

## Figures and Tables

**Table 1:** Reuse pit water and <4 mm sieved sediment chemical characteristics were collected during the December 2019 sampling date.

Constituent	Concentration
Water	
Sodium, mg L^−1^	42.5
Ammonium, mg N L^−1^	0.37
Potassium, mg L^−1^	118
Magnesium, mg L^−1^	10.8
Calcium, mg L^−1^	31.2
Chloride, mg L^−1^	81.6
Nitrite, mg N L^−1^	0.035
Nitrate, mg N L^−1^	4.89
Phosphate, mg P L^−1^	4.34
Sulfate, mg S L^−1^	18.7
pH	8.23
Alkalinity, mg CaCO_3_ L^−1^	141
DOC, mg C L^−1^	35.2
TDN, mg N L^−1^	8.94
Organic N, mg N L^−1^	3.64
Reuse pit: Chlortetracycline, µg L^−1^	<0.008
Reuse pit: Lincomycin, µg L^−1^	0.76
Reuse pit: Monensin, µg L^−1^	1.10
Reuse pit: Sulfadimethoxine, µg L^−1^	0.48
Sediment	
Total C, mg g^−1^	26.1 (1.28) ^a^
Total N, mg g^−1^	2.98 (0.12) ^a^
C/N, mg mg^−1^	8.77
KCl Extr. NH_4_^+^, µg N gdw^−1^	33.4 (7.6) ^b^
KCl Extr. NO_3_^−^ + NO_2_^−^, µg N gdw^−1^	1.83 (1.53) ^b^
KCl Extr. NO_2_^−^, µg N gdw^−1^	0.59 (0.1) ^b^

^a^ Mean values of triplicate CHN analyses of <4 mm sieved, dried, ground sediment; parentheses include standard deviation. ^b^ Mean values of triplicate 2M KCl extraction bottles containing 90 g KCl and 30 g wet sediment; parentheses include standard deviation. gDW, grams dry weight sediment.

**Table 2:** Reactant and product ions, cone voltages, collision energies, and retention times.

Compound	Parent Ion (*m*/*z*)	Product Ion (*m*/*z*)	Cone Voltage (V)	Collision Energy (eV)	Retention Time (min)
**Sulfamethazine-phenyl-13C8**	285.10	123.95	30	25	11.04
**Doxycycline**	445.05	428.05	29	19	12.34
**Demeclocycline**	464.9	447.9	27	17	11.50
**Sulfachloropyridazine**	285.0	155.95	24	15	11.42
**Chlortetracycline (total)**	478.9	444.0	28	20	12.05
**Lincomycin**	407.0	126.0	38	25	8.94
**Monensin (ammonium adduct)**	688.1	635.15	22	17	17.01
**Monensin (sodium adduct)**	693.7	675.7	22	25	17.01
**Sulfadimethoxine**	311.05	155.95	28	20	12.55
**Sulfamethazine**	279.1	155.95	30	18	11.04
**Tylosin**	916.9	174.2	50	35	12.43

**Table 3:** Concentrations of antibiotics in stock solutions and pre-incubation slurries across microcosm time points.

Sample ID	Collection Date	Chlortetracycline (µg L^−1^)	Lincomycin (µg L^−1^)	Monensin (µg L^−1^)	Sulfadimethoxine (µg L^−1^)
Antibiotic Mix C0 Stock ^a^	12/18/2019	<0.008	0.16	<0.033	<0.013
Antibiotic Mix C1 Stock ^a^	12/18/2019	<0.008	27.4	7.91	8.04
Antibiotic Mix C2 Stock ^a^	12/18/2019	2.87	339	111	144
Antibiotic Mix C3 Stock ^a^	12/18/2019	35.5	1682	324	631
Preincubation Slurry ^b^	12/18/2019	0.559 (0.549)	0.031 (0.022)	0.133 (0.06)	0.119 (0.087)
NF AB C0-TF ^c^	1/10/2020	<0.008	<0.027	<0.033	<0.013
NF AB C1-TF ^c^	1/10/2020	<0.008	0.586 (0.302)	<0.033	0.084 (0.042)
NF AB C2-TF ^c^	1/10/2020	<0.008	6.30 (4.38)	<0.033	1.58 (0.594)
NF AB C3-TF ^c^	1/10/2020	<0.008	57.7 (9.90)	0.044 (0.009)	3.91 (1.75)
DNF AB C0-TF ^d^	1/8/2020	0.081 (0.055)	0.045 (0.017)	<0.033	0.000
DNF AB C1-TF ^d^	1/8/2020	<0.008	12.2 (0.643)	2.22 (0.225)	1.87 (0.04)
DNF AB C2-TF ^d^	1/8/2020	0.063 (0.018)	139 (15)	24.1 (10.7)	29.5 (1.83)
DNF AB C3-TF ^d^	1/8/2020	0.462 (0.071)	681 (55.3)	182 (17.8)	243 (25)

^a^ Stock solutions containing a mixture of veterinary antibiotics with increasing concentrations C0, C1, C2, and C3. ^b^ Mean values of triplicate reuse pit sediment and water slurries with no additional antibiotics added and incubated for 10 min prior to collection; parentheses include standard deviation. ^c^ Mean values of samples collected from nitrification (NF) sample bottles at the end of the incubation period (TF); parentheses include standard deviation. ^d^ Mean values of samples collected from denitrification (DNF) sample bottles at the end of the incubation period (TF); parentheses include standard deviation.

**Table 4:** Mean values and standard deviations of physiochemical parameters measured during the mesocosm experiment.

Treatment	DO (mg L^−1^)	Conductivity (μS cm^−1^)	ORP (mV)	Temperature (°C)	pH Range (min–max)	DOC (mg L^−1^)
Control	3.9 ± 1.66	681.9 ± 10.27	90.33 ± 35.47	27.6 ± 1.23	6.55–7.29	4.29 ± 0.52
Control Antibiotics	1.76 ± 2.72	670.43 ± 11.9	−90.9 ± 129.84	27.63 ± 1.37	6.57–7.31	79.51 ± 17.43
FTW	0.8 ± 0.16	873.71 ± 41.98	5.39 ± 70.96	26.23 ± 0.79	5.79–7.69	13.14 ± 1.86
FTW + Antibiotics	0.24 ± 0.2	882.38 ± 30.92	−236.4 ± 97.99	25.6 ± 1.08	6.37–7.22	76.06 ± 6.2

**Table 5:** Mass values and standard deviations of VAs in water on different sampling days of the mesocosm experiment. Standard deviations are denoted in parentheses. Detection limits for each VA were as follows: chlortetracycline (<0.0008 μg L^−1^), lincomycin (<0.027 μg L^−1^), monensin (<0.033 μg L^−1^), sulfamethazine (<0.013 μg L^−1^).

VA	Day	Control (mg)	Control-VA (mg)	FTW (mg)	FTW-VA (mg)
Chlortetracycline	1	<0.008	<0.008	<0.008	4.2 (±0.7)
	5	<0.008	<0.008	<0.008	1.1 (±0.4)
	10	<0.008	<0.008	<0.008	0.9 (±0.2)
Lincomycin	1	<0.027	106.2 (±28.7)	<0.027	208.8 (±39.0)
	5	<0.027	105.5 (±29.7)	<0.027	254.3 (±47.6)
	10	<0.027	108.1 (±25.9)	<0.027	364.5 (±59.7)
Monensin	1	<0.033	100.4 (±13.2)	<0.033	110.4 (±7.7)
	5	<0.033	176.6 (±53.7)	<0.033	103.0 (±19.2)
	10	<0.033	347.8 (±18.2)	<0.033	250.4 (±76.4)
Sulfamethazine	1	<0.013	265.1 (±12.4)	<0.013	185.2 (±20.4)
	5	<0.013	108.2 (±53.9)	<0.013	287.0 (±49.1)
	10	<0.013	57.3 (±54.5)	<0.013	251.6 (±55.9)

## Data Availability

The data presented in this study are openly available at: Repert, D.A.; Reed, A.P.; Smith, R.L.; Bartelt-Hunt, S.; Messer, T.; Russell, M.; Snow, D.; Underwood, J.C. Biogeochemical and microbial data from microcosm experiments using wetland sediment to investigate the influence of antibiotics and a nitrification inhibitor in agricultural run-off on N-cycling processes, 2019–2020: U.S. Geological Survey data release, 2024. https://doi.org/10.5066/P9H1X6DR. Microcosm biogeochemical, microbial community, and N-cycling gene abundance data are available in USGS *ScienceBase* data release https://doi.org/10.5066/P9H1X6DR (Repert et al., 2024). Microbial sequence data are deposited in the NCBI Sequence Read Archive (https://www.ncbi.nlm.nih.gov/sra) under BioProject no. PRJNA1104208 and accession numbers SAMN41071481-SAMN41071559. Messer, T. (6 May 2024). Influence of Four Veterinary Antibiotics on Constructed Treatment Wetland Nitrogen Transformation. Retrieved from osf.io/8gbam.

## Supplementary Materials

The following supporting information can be downloaded at https://www.mdpi.com/article/10.3390/toxics12050346/s1, Figure S1. Method preparation of antibiotic mixture solutions added to denitrification and nitrification assay bottles; Figure S2. Method details for anaerobic denitrification potential assays; Figure S3. Method details for aerobic nitrification potential assays; Figure S4. Time course of (A) nitrate production, (B) nitrite production, and (C) ammonium consumption by nitrification in oxic sediment slurries amended with ammonium (14 mg N L−1) and a mixture of 4 antibiotics; Figure S5. Time course of nitrous oxide (N2O-N) production by denitrification in anoxic sediment slurries amended with nitrate (14 mg N L−1), acetylene, and a mixture of 4 antibiotics; Figure S6. Chlortetracycline relationships between dissolved oxygen, conductivity, ORP, water temperature, pH, and DOC in the mesocosm experiment; Figure S7. Lincomycin relationships between dissolved oxygen, conductivity, ORP, water temperature, pH, and DOC in the mesocosm experiment; Figure S8. Monensin relationships between dissolved oxygen, conductivity, ORP, water temperature, pH, and DOC in the mesocosm experiment; Figure S9. Sulfamethazine relationships between dissolved oxygen, conductivity, ORP, water temperature, pH, and DOC in the mesocosm experiment; Table S1. Recipe for artificial water used for December 2019 reuse pit denitrification and nitrification assays; Table S2. Antibiotic concentrations from reuse pit background sample, stock solutions, preincubation slurries, and final time points from denitrification and nitrification incubations collected December 2019 and January 2020; Table S3. Areal concentrations for VAs in plant biomass. Table S4. VA mass values recovered from water. The original contributions presented in this study are included in the article/Supplementary Materials, further inquiries can be directed to the corresponding author. References [97,98,99] are cited in the Supplementary Material.

## Abbreviations

VA	Veterinary Antibiotic
CAFO	Confined Animal Feeding Operation
FTW	Floating Treatment Wetland
USDA	United States Department of Agriculture
USMARC	United States Meat Animal Research Center
USGS	United States Geological Survey
DOC	Dissolved Organic Carbon
TDN	Total Dissolved Nitrogen
ORP	Oxidation Reduction Potential
DO	Dissolved Oxygen
HDPE	High-Density Polyethylene
LC-MS/MS	Liquid Chromatography–Mass Spectrometry/Mass Spectrometry
