# Supplementary material for: Influence of Four Veterinary Antibiotics on Constructed Treatment Wetland Nitrogen Transformation
Source: Toxics. 2024 May 8;12(5):346. doi: 10.3390/toxics12050346 (PMC11125918; doi:10.3390/toxics12050346)
Supplement: Supplementary file 1 [file toxics-12-00346-s001.zip › toxics-2926726-supplementary.pdf]

Contents of this file

Figure S1. Method preparation of antibiotic mixture solutions added to denitrification and nitrification assay bottles.

Figure S2. Method details for anaerobic denitrification potential assays.

Figure S3. Method details for aerobic nitrification potential assays.

Figure S4. Time course of (A) nitrate production, (B) nitrite production, and (C) ammonium consumption by nitrification in oxic sediment slurries amended with ammonium (14 mg N L^{-1}) and a mixture of 4 antibiotics.

Figure S5. Time course of nitrous oxide (N_2O-N) production by denitrification in anoxic sediment slurries amended with nitrate (14 mg N L^{-1}), acetylene, and a mixture of 4 antibiotics.

Figure S6. Chlortetracycline relationships between dissolved oxygen, conductivity, ORP, water temperature, pH, and DOC in the mesocosm experiment.

Figure S7. Lincomycin relationships between dissolved oxygen, conductivity, ORP, water temperature, pH, and DOC in the mesocosm experiment.

Figure S8. Monensin relationships between dissolved oxygen, conductivity, ORP, water temperature, pH, and DOC in the mesocosm experiment.

Figure S9. Sulfamethazine relationships between dissolved oxygen, conductivity, ORP, water temperature, pH, and DOC in the mesocosm experiment.

Table S1. Recipe for artificial water used for December 2019 Reuse Pit denitrification and nitrification assays.

Table S2. Antibiotic concentrations from Reuse Pit background sample, stock solutions, preincubation slurries, and final timepoints from denitrification and nitrification incubations collected December 2019 and January 2020.

Table S3. Areal concentrations for VAs in plant biomass.

Table S4. VA mass values recovered from water.

References

1. Brooks, M.H.; Smith, R.L.; Macalady, D.L. Inhibition of existing denitrification enzyme activity by chloramphenicol. *Appl. Environ. Microbiol.* **1992**, *58*, 1746–1753, https://doi.org/10.1128/aem.58.5.1746-1753.1992.

2. Smith, R.L., Baumgartner, L.K., Miller, D.N., Repert, D.A., and Böhlke, J.K. Assessment of nitrification potential in groundwater using short term, single-well injection experiments. *Microb. Ecol.* **2006**, *51*, 22–35, https://doi.org/10.1007/s00248-004-0159-7.

3. Smith, R.L.; Repert, D.A.; Stoliker, D.L.; Kent, D.B.; Song, B.; LeBlanc, D.R.; McCobb, T.D.; Böhlke, J.K.; Hyun, S.P.; Moon, H.S. Seasonal and Spatial Variation in the Location and Reactivity of a Nitrate-Contaminated Groundwater Discharge Zone in a Lakebed. *J. Geophys. Res. Biogeosciences* **2019**, *124*, 2186–2207, https://doi.org/10.1029/2018jg004635.

Funding

This material is based upon work supported by the United States Department of Agriculture under grant no. 2019-67019-29487. This project was also supported with funds by the United States Hatch multistate capacity funding grant (W-4045). Any opinions, findings, conclusions, or recommendations expressed in this publication are those of the authors and do not necessarily reflect the view of the U.S. Department of Agriculture. Any use of trade, firm, or product names is for descriptive purposes only and does not imply endorsement by the U.S. Government.

Data Sources

Data presented in this document and additional geochemical and hydrologic data can be found in:

U.S. Geological Survey (USGS) data release: Repert, D.A., Reed, A.P., Smith, R.L., Bartelt-Hunt, S., Messer, T., Russell, M., Snow, D., and Underwood, J.C., 2024, Biogeochemical and microbial data from microcosm experiments using wetland sediment to investigate the influence of antibiotics and a nitrification inhibitor in agricultural run-off on N-cycling processes, 2019-2020: U.S. Geological Survey data release, https://doi.org/10.5066/P9H1X6DR

Microcosm biogeochemical, microbial community, and N-cycling gene abundance data are available in USGS ScienceBase data release https://doi.org/10.5066/P9H1X6DR (Repert et al., 2024). Mi-crobial sequence data are deposited in the NCBI Sequence Read

Archive (https://www.ncbi.nlm.nih.gov/sra) under BioProject no. PRJNA1104208 and accession numbers SAMN41071481 - SAMN41071559.

Messer, T. (2024, May 6). Influence of Four Veterinary Antibiotics on Constructed Treatment Wetland Nitrogen Transformation. Retrieved from osf.io/8gbam.

Antibiotic Mixture Preparation:

- Prepared aerobically
- Concentration 3 → 1,000 μg of each antibiotic in 1 L artificial water
- Concentration 2 → 140 mL 1000 μg L^{-1} in 560 mL artificial water
- Concentration 1 → 14 mL 1000 μg L^{-1} in 686 mL artificial water
- Concentration 0 → artificial water only
- Half of each solution was placed in anaerobic glove bag to deoxygenate for the anerobic denitrification potential experiments
- Half of each solution was kept aerobic for the aerobic nitrification potential experiments

Antibiotic	Mode of Action	Target concentration (μg L^{-1})	Actual Concentration (μg L^{-1})
Chlortetracycline	Bacteriostatic (protein synthesis inhibitor)	1000	1010
Lincomycin	Bacteriostatic (protein synthesis inhibitor)	1000	1780
Sulfadimethoxine	Bacteriostatic (nucleotide synthesis inhibitor)	1000	1050
Monensin	Bacteriostatic (alter ion flow across cell membrane)	1000	1140

Figure S1. Method preparation of antibiotic mixture solutions added to denitrification and nitrification assay bottles, including targeted concentrations and actual concentrations based on weight measurements.

Anaerobic Denitrification Potential Experiments

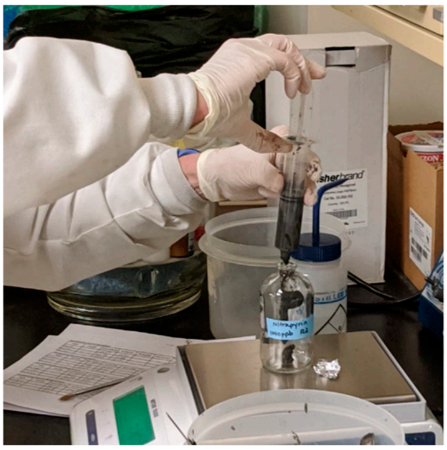

Microcosm setup:

- Weighed sieved sediment in bottles, transferred to anaerobic glove bag to add water with inhibitors
    - 12 grams wet sediment, 100 grams artificial water
    - 125 mL glass serum bottle, stoppered
- Removed from glove bag, flushed headspace with argon
- Placed on end-over-end rotator for 3 weeks at room temperature in the dark

After 3 weeks of inhibitor exposure:

- Flushed serum bottle headspace with argon
- Added nitrate stock for final concentration of 14 mg nitrate-N L^{-1}, 10 mL acetylene
- Measured nitrous oxide production over time via gas chromatography
    - Time course: every 20 min for 1 ½ hours
- Mixed end-over-end in the dark between time points

Analysis:

- HNU Gas Chromatograph with electron capture detector [1]
- Sievers Instruments Model 280i NOA [2]
- Dionex Model ICS-5000 [3]

Figure S2. Method details for anaerobic denitrification potential assays.

Aerobic Nitrification Potential Experiments

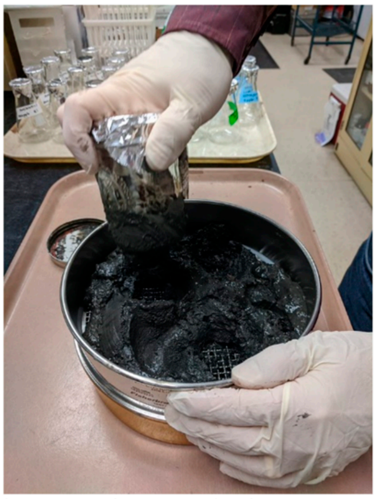

Microcosm setup:

- Weighed and combined sieved sediment and water with inhibitors in glass flasks aerobically
    - 12 grams sediment, 100 grams artificial water
    - 125 mL glass Erlenmeyer flasks
- Placed on reciprocal shaker table for 3 weeks at room temperature in the dark

After 3 weeks of inhibitor exposure:

- Added 14 mg ammonium-N L^{-1} to flasks, continued to shake on reciprocal table
- Collected 5 mL water + sediment at 8 – 12-hour intervals
- Water sample filtered and preserved for nitrate, nitrite, and ammonium analysis

Analysis:

- Sievers Instruments Model 280i NOA [2]
- Dionex Model ICS-5000 [3]

Figure S3. Method details for aerobic nitrification potential assays.

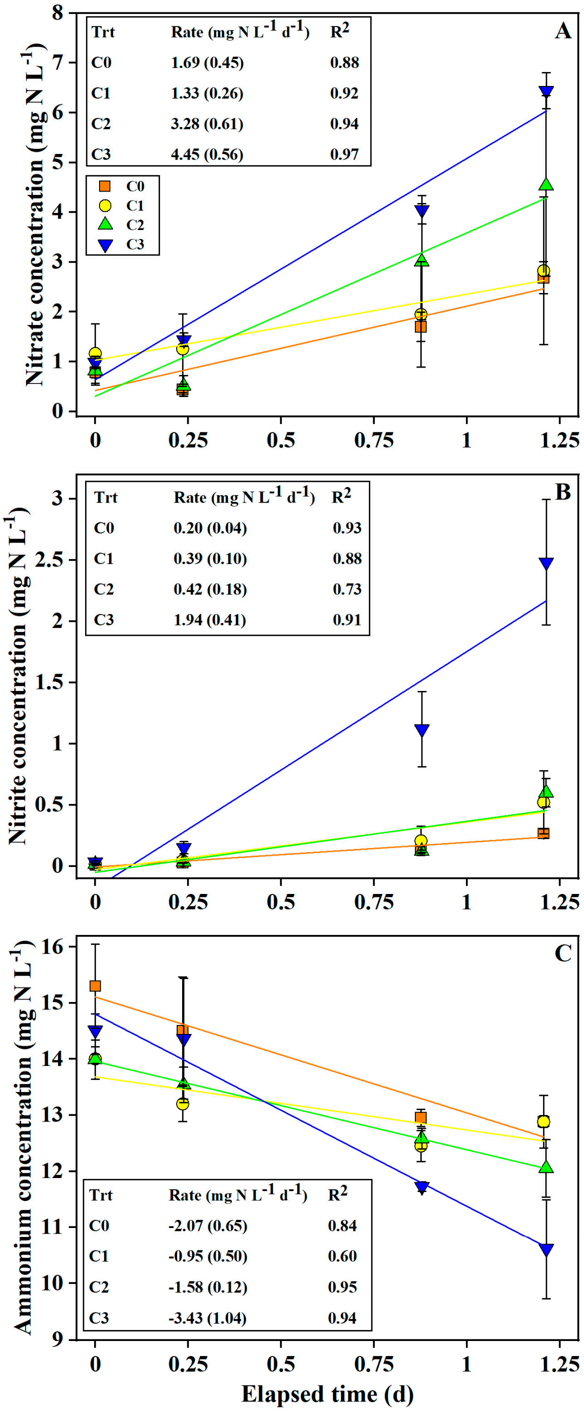

Figure S4. Time course of (A) nitrate production, (B) nitrite production, and (C) ammonium consumption by nitrification in oxic sediment slurries amended with ammonium (14 mg N L^{-1}) and a mixture of 4 antibiotics. The final concentrations for the respective antibiotics for C0, C1, C2, and C3 are shown in Table S3. Lines are best-fit

linear regressions used to derive rates of activity (inset). Data points are mean values of triplicate bottles; brackets enclose ± 1 standard deviation.

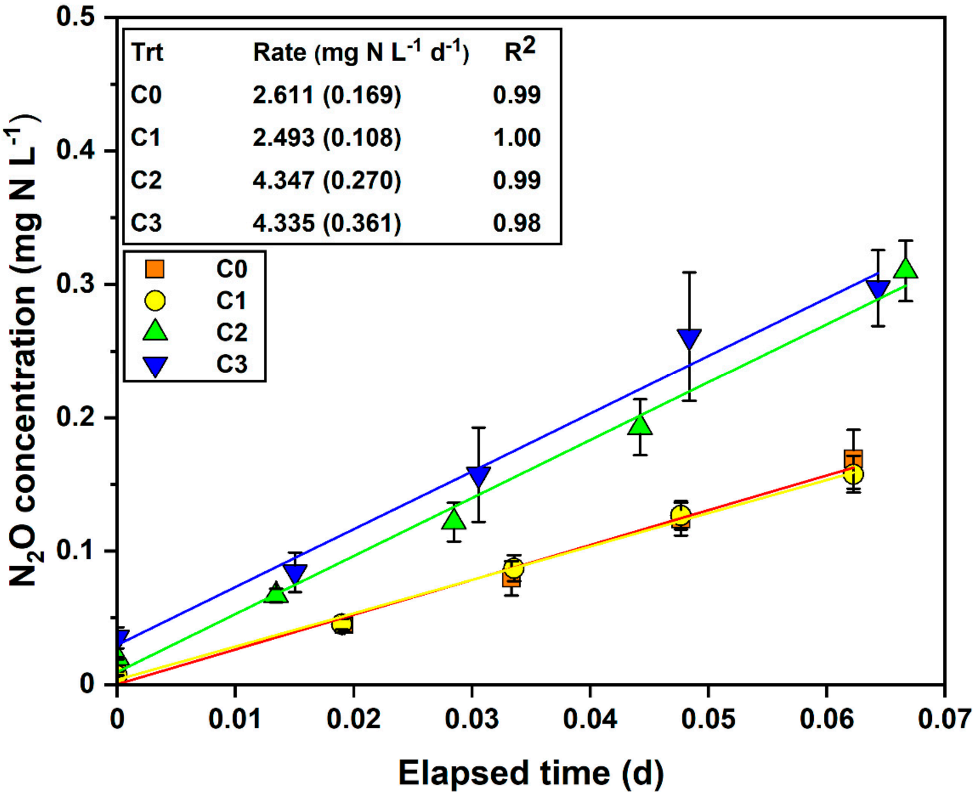

Figure S5. Time course of nitrous oxide (N_2O-N) production by denitrification in anoxic sediment slurries amended with nitrate (14 mg N L^{-1}), acetylene, and a mixture of 4 antibiotics, each at the indicated final concentration. The final concentrations for the respective antibiotics for C0, C1, C2, and C3 are shown in Table S3. Lines are best-fit linear regressions used to derive rates of activity (inset). Data points are mean values of triplicate bottles; brackets enclose ± 1 standard deviation [S3].

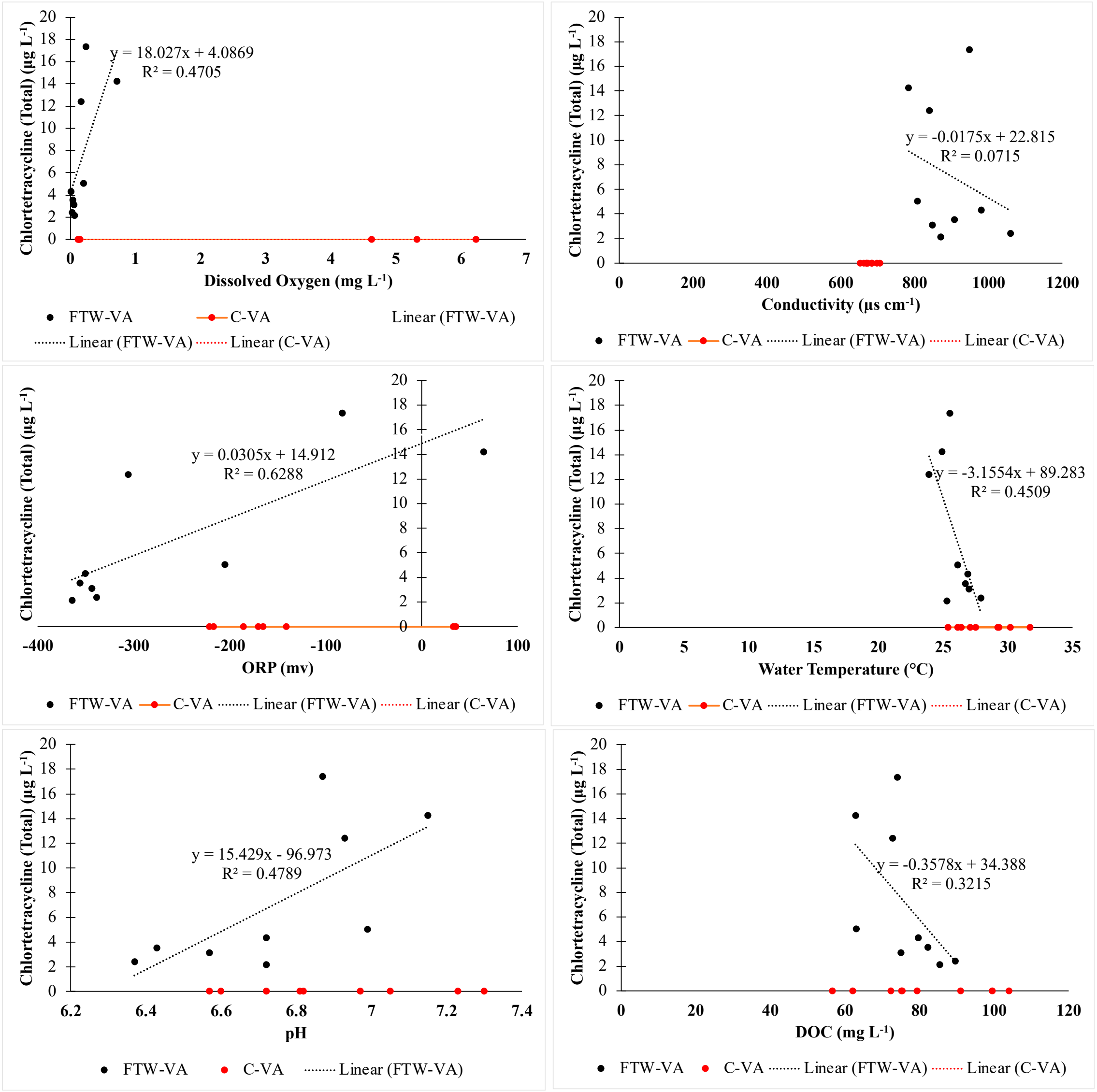

Figure S6. Chlortetracycline relationships between dissolved oxygen, conductivity, ORP, water temperature, pH, and DOC in the mesocosm experiment.

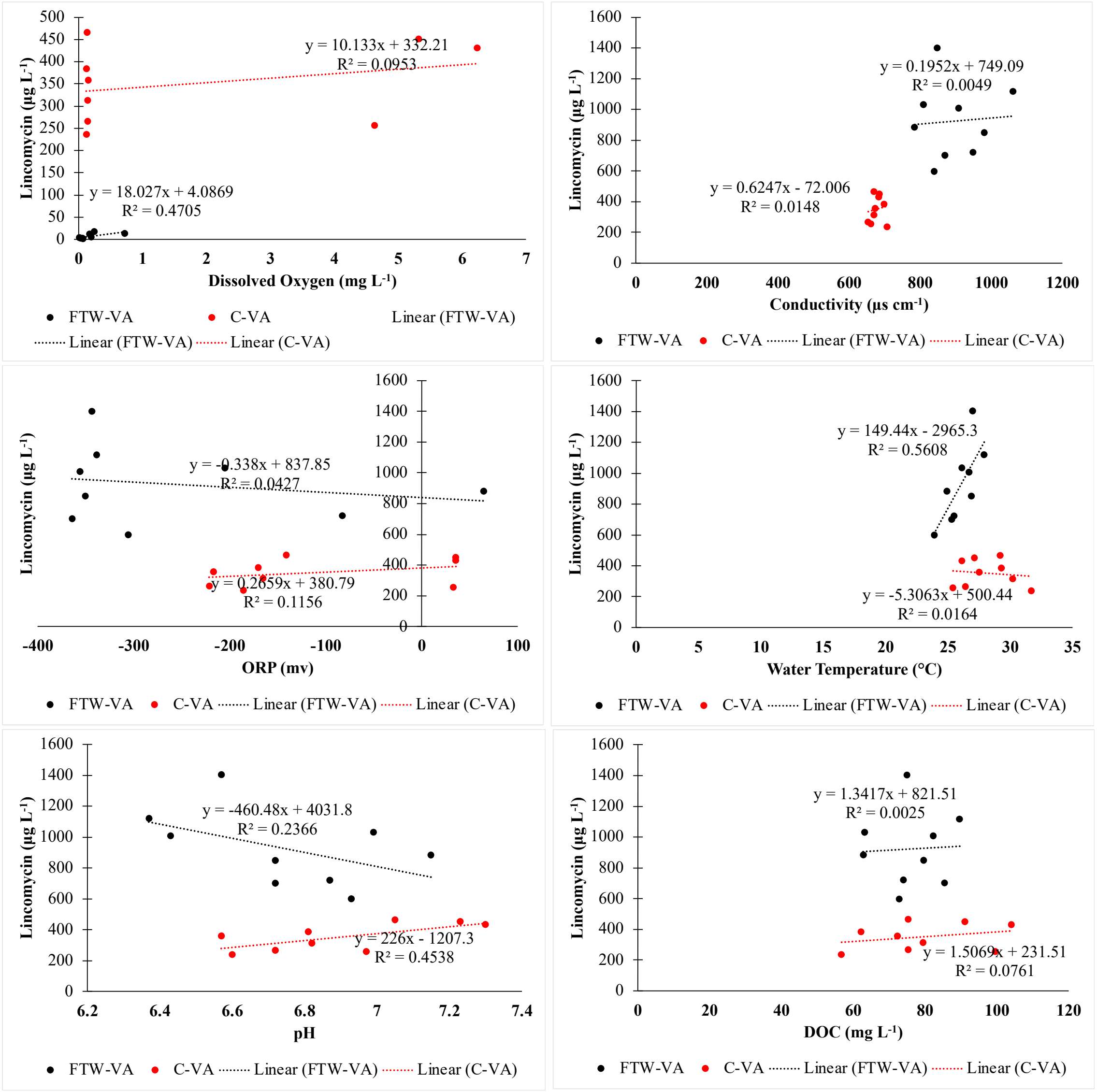

Figure S7. Lincomycin relationships between dissolved oxygen, conductivity, ORP, water temperature, pH, and DOC in the mesocosm experiment.

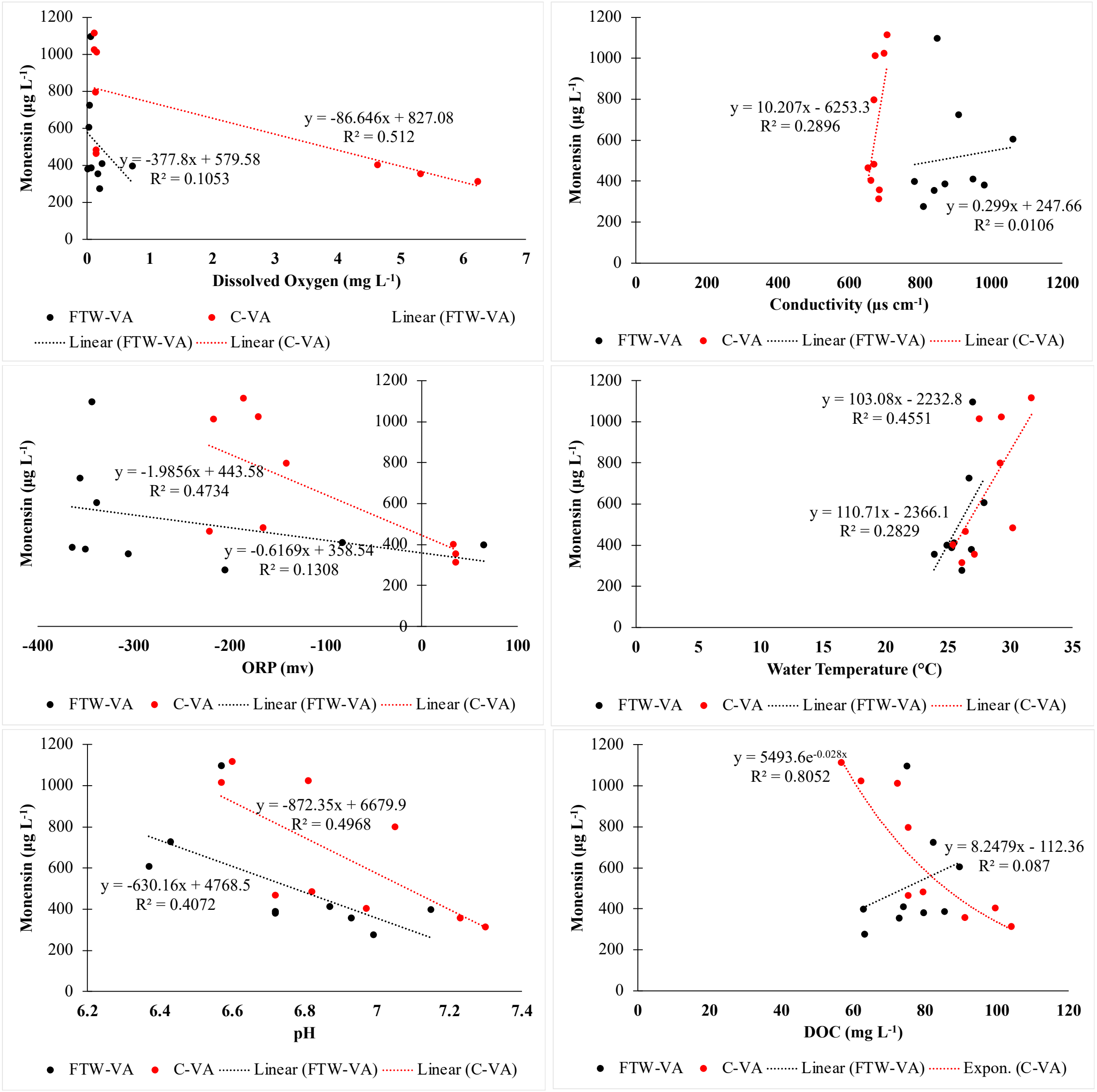

Figure S8. Monensin relationships between dissolved oxygen, conductivity, ORP, water temperature, pH, and DOC in the mesocosm experiment.

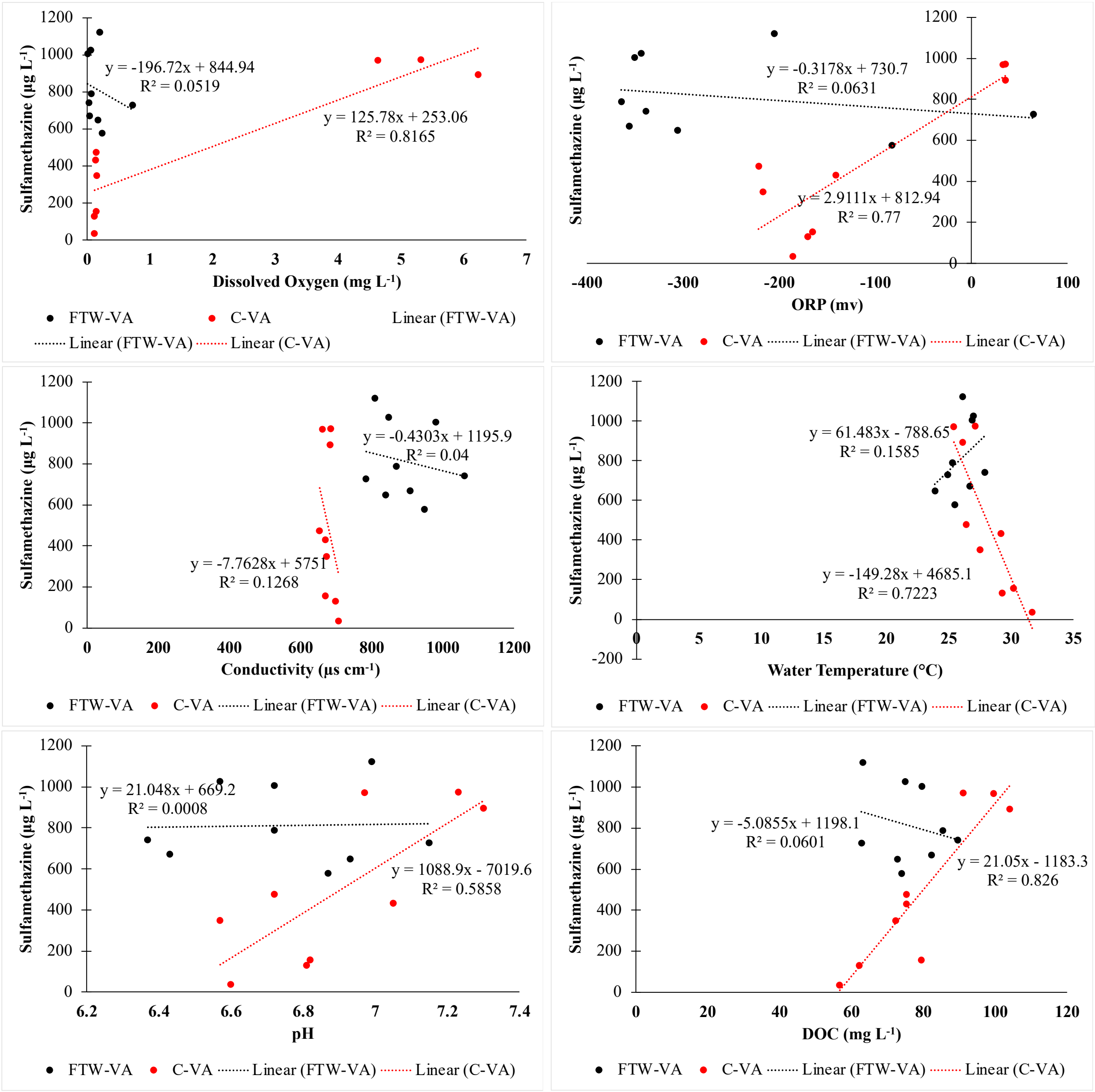

Figure S9. Sulfamethazine relationships between dissolved oxygen, conductivity, ORP, water temperature, pH, and DOC in the mesocosm experiment.

Table S1. Recipe for artificial water used for December 2019 Reuse Pit denitrification and nitrification assays. Concentrations were based on measured chemistry of Reuse Pit water collected in August 2019.

Chemical	mg L^{-1} added	Target Ions
$CaCl_2$	0.0368	Ca^{2+}, Cl^-
KH_2PO_4	0.0127	K^+, PO_4^3
$KHCO_3$	0.0984	K^+, HCO_3^-
$MgSO_4$*$7H_2O$	0.0291	Mg^{2+}, SO_4^{2-}
$NaHCO_3$	0.0212	Na^+, HCO_3^-

Table S2. Antibiotic concentrations from preincubation slurries and final timepoints from denitrification and nitrification incubations collected December 2019 and January 2020.

**Sample ID**	**Collection Date**	**Chlortetracycline ($\mu g\ L^{-1}$)**	**Lincomycin ($\mu g\ L^{-1}$)**	**Monensin ($\mu g\ L^{-1}$)**	**Sulfadimethoxine ($\mu g\ L^{-1}$)**
Preincubation Slurry R1[a]	12/18/2019	<0.008	<0.027	0.073	0.043
Preincubation Slurry R2[a]	12/18/2019	1.10	0.056	0.194	0.214
Preincubation Slurry R3[a]	12/18/2019	0.57	<0.027	0.131	0.100
NF AB C0 - TF R1[b]	1/10/2020	<0.008	<0.027	<0.033	<0.013
NF AB C0 - TF R2[b]	1/10/2020	<0.008	<0.027	<0.033	<0.013
NF AB C0 - TF R3[b]	1/10/2020	<0.008	<0.027	<0.033	<0.013
NF AB C1 - TF R1[b]	1/10/2020	<0.008	0.731	<0.033	0.121
NF AB C1 - TF R2[b]	1/10/2020	<0.008	0.239	<0.033	0.039
NF AB C1 - TF R3[b]	1/10/2020	<0.008	0.788	<0.033	0.091
NF AB C2 - TF R1[b]	1/10/2020	<0.008	9.34	<0.033	2.08
NF AB C2 - TF R2[b]	1/10/2020	<0.008	1.27	<0.033	0.925
NF AB C2 - TF R3[b]	1/10/2020	<0.008	8.29	<0.033	1.73
NF AB C3 - TF R1[b]	1/10/2020	<0.008	64.7	0.053	5.93
NF AB C3 - TF R2[b]	1/10/2020	<0.008	62.1	0.036	2.99
NF AB C3 - TF R3[b]	1/10/2020	<0.008	46.4	0.042	2.81
DNF AB C0 - TF R1[c]	1/8/2020	0.143	0.047	<0.033	<0.013
DNF AB C0 - TF R2[c]	1/8/2020	0.063	<0.027	<0.033	<0.013
DNF AB C0 - TF R3[c]	1/8/2020	0.036	0.06	<0.033	<0.013
DNF AB C1 - TF R1[c]	1/8/2020	<0.008	12.9	2.28	1.84
DNF AB C1 - TF R2[c]	1/8/2020	<0.008	11.6	1.98	1.91
DNF AB C1 - TF R3[c]	1/8/2020	<0.008	12.2	2.41	1.85

DNF AB C2 - TF R1[c]	1/8/2020	0.073	157	36.4	30.7

[a]Reuse Pit sediment and water slurries containing no antibiotics and incubated for 10 minutes prior to collection.

[b]Samples collected from nitrification (NF) sample bottles at the end of the incubation period (TF).

[c]Samples collected from denitrification (DNF) sample bottles at the end of the incubation period (TF).

Table S3. Areal concentrations for VAs in plant biomass, calculated using the total FTW surface area ($0.36 m^2$) and the recovered mass (mg) of each VA in the below (BS) and above surface (AS) biomass. Standard deviations (SD) are reported in the column following each VA.

**Average aerial VA removal in plant tissue (mg m^{-2})**								
	**Chlortetracycline**	**SD**	**Lincomycin**	**SD**	**Monensin**	**SD**	**Sulfamethazine**	**SD**
**FTWA (AS)**	0.0	0.0	0.6	0.9	0.0	0.0	0.2	0.2
**FTWA (BS)**	122.8	60.9	34.7	20.6	8.4	5.4	25.5	11.1

Table S4. Mass values of VAs recovered from water.

**VAs recovered in FTWA water (mg)**				
	**Chlortetracycline**	**Monensin**	**Lincomycin**	**Sulfamethazine**
**FTWA1**	1.0	430.0	336.2	314.6
**FTWA2**	0.8	350.4	189.7	232.4
**FTWA3**	1.1	313.1	225.3	207.9
**Ave**	0.9	364.5	250.4	251.6
